# OMSV enables accurate and comprehensive identification of large structural variations from nanochannel-based single-molecule optical maps

**DOI:** 10.1186/s13059-017-1356-2

**Published:** 2017-12-01

**Authors:** Le Li, Alden King-Yung Leung, Tsz-Piu Kwok, Yvonne Y. Y. Lai, Iris K. Pang, Grace Tin-Yun Chung, Angel C. Y. Mak, Annie Poon, Catherine Chu, Menglu Li, Jacob J. K. Wu, Ernest T. Lam, Han Cao, Chin Lin, Justin Sibert, Siu-Ming Yiu, Ming Xiao, Kwok-Wai Lo, Pui-Yan Kwok, Ting-Fung Chan, Kevin Y. Yip

**Affiliations:** 1 Department of Computer Science and Engineering, The Chinese University of Hong Kong, Shatin, New Territories Hong Kong; 2 School of Life Sciences, The Chinese University of Hong Kong, Shatin, New Territories Hong Kong; 3 Cardiovascular Research Institute, University of California San Francisco, San Francisco, California USA; 4 Department of Anatomical and Cellular Pathology, The Chinese University of Hong Kong, Shatin, New Territories Hong Kong; 5 Department of Computer Science, The University of Hong Kong, Pokfulam, Hong Kong; 6 BioNano Genomics, San Diego, California USA; 7 School of Biomedical Engineering, Science and Health Systems, Drexel University, Philadelphia, Pennsylvania USA; 8 Institute for Human Genetics, University of California San Francisco, San Francisco, California USA; 9 Hong Kong Bioinformatics Centre, The Chinese University of Hong Kong, Shatin, New Territories Hong Kong; 10 Hong Kong Institute of Diabetes and Obesity, The Chinese University of Hong Kong, Shatin, New Territories Hong Kong; 11 CUHK-BGI Innovation Institute of Trans-omics, The Chinese University of Hong Kong, Shatin, New Territories Hong Kong

**Keywords:** Optical mapping, Nanochannel, Single-molecule analysis, Structural variation

## Abstract

We present a new method, OMSV, for accurately and comprehensively identifying structural variations (SVs) from optical maps. OMSV detects both homozygous and heterozygous SVs, SVs of various types and sizes, and SVs with or without creating or destroying restriction sites. We show that OMSV has high sensitivity and specificity, with clear performance gains over the latest method. Applying OMSV to a human cell line, we identified hundreds of SVs >2 kbp, with 68 % of them missed by sequencing-based callers. Independent experimental validation confirmed the high accuracy of these SVs. The OMSV software is available at http://yiplab.cse.cuhk.edu.hk/omsv/.

**Electronic supplementary material:**

The online version of this article (doi:10.1186/s13059-017-1356-2) contains supplementary material, which is available to authorized users.

## Background

Structural variations (SVs), defined as genomic alterations involving segments larger than 1 kbp [1], are prevalent in human genomes. They represent characteristic differences among human populations [2], and are associated with various diseases [3, 4].

Current sequencing technologies, including second-generation and commercial third-generation sequencing platforms, produce sequencing reads from a hundred to tens of thousands of base pairs only, making it challenging to study long repetitive regions and complex structural rearrangements. For instance, some large insertions cannot be contained in a single read, and their detection requires either sequence assembly [5] or reference alignment [6, 7], with the help of paired-end or mate-pair sequencing with large insert sizes [8, 9]. In general, these methods are not ideal for detecting large SVs accurately and comprehensively, especially SVs that involve long DNA sequences not present in the reference sequence [10, 11].

**Fig. 1:**
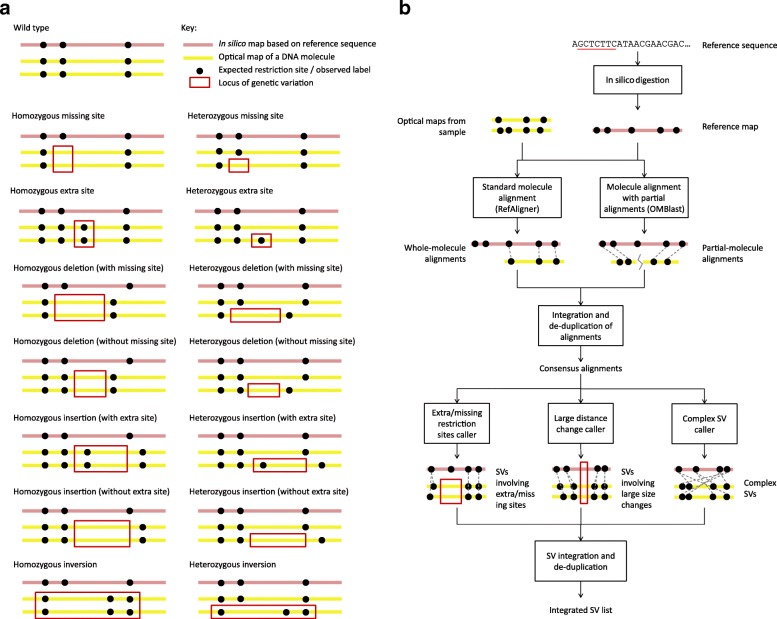
Underlying concepts of OMSV. **a** Different types of genetic variations and their idealized appearance patterns on optical maps. Real optical-mapping data contain various types of errors that make these patterns less apparent. Inversions are shown as an example type of complex SVs, while OMSV can also detect translocations and copy number variations. **b** The overall OMSV pipeline for identifying SVs from optical maps. Optical maps from a study sample are aligned to the reference map using two different aligners. Their results are integrated to form a single list of consensus alignments, which are then passed to three SV-calling modules to identify different types of SVs. SV, structural variation

Optical mapping (OM) [12] is a promising alternative technology that provides structural information about individual long DNA molecules. In nanochannel-based OM [13, 14], DNA molecules are digested by a nicking endonuclease to create single-strand nicks, which are then repaired with fluorescent dye conjugated nucleotides. The resulting DNA molecules are linearized in nanochannels and imaged using high-resolution fluorescent microscopy (Additional file 1: Figure S1). The final outputs are the optical maps, which record restriction site label locations on each DNA molecule. SVs can be identified by comparing the observed label pattern with the expected pattern based on the reference sequence (Fig. 1 a). For example, two sites significantly farther apart on an optical map than their corresponding locations on the reference could indicate an insertion.

Due to the much longer length of optical maps (up to 1 Mbp) compared to sequencing reads, OM is very powerful in SV discovery [13, 15–18]. Current high-throughput OM methods can produce optical maps for a hundred thousand molecules within a few hours, at an average size of several hundred kilobase pairs per molecule. These molecules can be full-length DNA derived from species with a small genome, or fragments of very long DNA molecules such as human chromosomes.

Analyzing optical maps is non-trivial due to various types of error in the data [19, 20]. False positives (false labels observed but not from true restriction sites) can occur due to non-specific enzymatic cuts or DNA breakage. False negatives (true restriction sites not observed on the optical maps) can occur due to incomplete enzyme digestion. Sizing errors (deviations between measured and actual distance between two restriction sites on an optical map) can occur due to DNA fragments that are over-stretched or not completely linearized. Finally, labels of close restriction sites may merge into a single label in the observed data due to limitations in imaging resolution. As a result of all these error types, specialized methods have been proposed for various computational tasks related to the analysis of optical maps, including error modeling [19, 21], molecule alignment [18, 20, 22–24], de novo and reference-assisted assembly [20, 25, 26], and detection of SVs [13, 17, 18, 27].

Existing methods for calling SVs from optical maps have several major limitations (Additional file 1: Table S1). First, most of them require a de novo assembly of the optical maps or the construction of a consensus map [13, 15, 18], making the accuracy of SV calls dependent on the reliability of these difficult procedures. Second, none of the current methods can simultaneously (1) detect both homozygous and heterozygous SVs, (2) handle SVs of a wide range of sizes, and (3) evaluate SV probabilities based on a formal error model and the optical maps that support or do not support the SVs. Besides, almost none of the existing methods have made their software publicly available, which hampers the widespread use of OM in studying SVs.

Here we describe a comprehensive SV-calling pipeline and corresponding open-source software, OMSV (available at the supplementary website, http://yiplab.cse.cuhk.edu.hk/omsv/, under the MIT license), which overcomes these limitations. We demonstrate the effectiveness of OMSV using both simulations and optical maps produced from a family trio. In addition, we show that when OMSV was applied to detect SVs in a human cell line, many of our detected SVs were missed by typical sequencing-based SV callers. Some of our detected SVs were experimentally tested using DNA isolated from the cell line, and most of them were successfully validated. Finally, we describe how OMSV can combine optical maps and sequencing data to identify precise SV break points and uncover novel sequences involved in the SVs.

## Results

### The OMSV pipeline

OMSV contains two main steps (Fig. 1 b, “Methods”). In the first step, it aligns optical maps to the reference map, which is deduced from the reference sequence and the recognition motif of the nicking enzyme by in silico digestion. Two different aligners are used, namely RefAligner [24], which can efficiently align optical maps highly similar to the reference, and OMBlast [22], which can handle more complex genomic rearrangements by split-aligning a single optical map to multiple regions on the reference. The alignment results from the two aligners are integrated to form a single set of consensus alignments. In the second step of OMSV, these alignments are passed to three separate SV-calling modules for three corresponding types of SVs: (1) SVs involving the creation or removal of restriction sites, (2) SVs involving large distance changes between restriction sites, and (3) more complex SVs, such as inversions and translocations. SVs identified from these modules are then integrated and de-duplicated to form a final list of SVs.

**Fig. 2:**
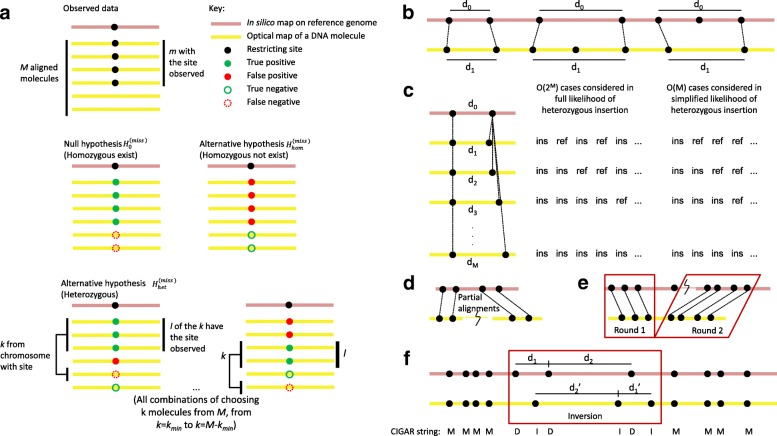
Illustration of methods used by OMSV for identifying SVs from optical maps. **a** The three hypotheses compared in the procedure for detecting missing restriction sites. **b** Comparing the distance between two restriction sites on the reference and the corresponding observed labels on the optical maps, for detecting large SVs. **c** Simplification of the likelihood function for the heterozygous insertion hypothesis. In the full likelihood function, each optical map could come from the chromosome with the reference allele (ref) or the insertion allele (ins), and all combinations are considered. In the simplified likelihood function, only the *k* optical maps with the largest distance between the two nicking site labels are considered to have the insertion, and all values of *k* are considered. In this illustration, the minimum number of optical maps supporting each allele, *k*_min_, is set to 0. **d** SVs that require partial alignments to identify. **e** Translocations and large inversions can be identified by two-round split alignments. **f** Medium-sized inversions are identified by looking for regions with a reverse palindromic CIGAR string (DIDIDI in this example) with matched segment sizes when reversed (*d*_1_ with *d*1′ and *d*_2_ with *d*2′ in this example). ins, insertion allele, ref, reference allele, SV, structural variation

In the SV-calling modules, a formal error model is used to compare the likelihoods of the reference genotype (i.e., no SVs), homozygous SVs, and heterozygous SVs. An SV is called only if a set of stringent criteria are satisfied (Fig. 2, “Methods”).

### Simulations confirm the effectiveness of OMSV

To test the effectiveness of OMSV, we generated simulated OM data from artificial haploid and diploid human genomes, by introducing various types of genetic variants into the reference genome hg38 followed by simulating noisy optical maps with all types of error (“Methods”). We defined a default error setting, and additional settings that covered a wide range of false positive and false negative rates of nicking sites and depths of coverage, leading to a total of 28 sets of simulated OM data (Additional file 1: Tables S2 and S3). In the original paper that describes the nanochannel-based OM method [13], the false positive and false negative rates were reported to be 21 % and 4 %, respectively. According to our experience, the current systems have around 10 % false negative labels and one false positive label per 100 kbp. In the default error setting, we set these parameters to slightly higher values to test OMSV’s ability to handle noisy data (Additional file 1: Table S2).

Next, we applied OMSV to identify SVs from these simulated OM data sets, and compared the results to the actual lists of synthesized SVs to determine OMSV’s precision (fraction of called SVs that are correct) and recall (fraction of simulated SVs correctly called by OMSV).

**Fig. 3:**
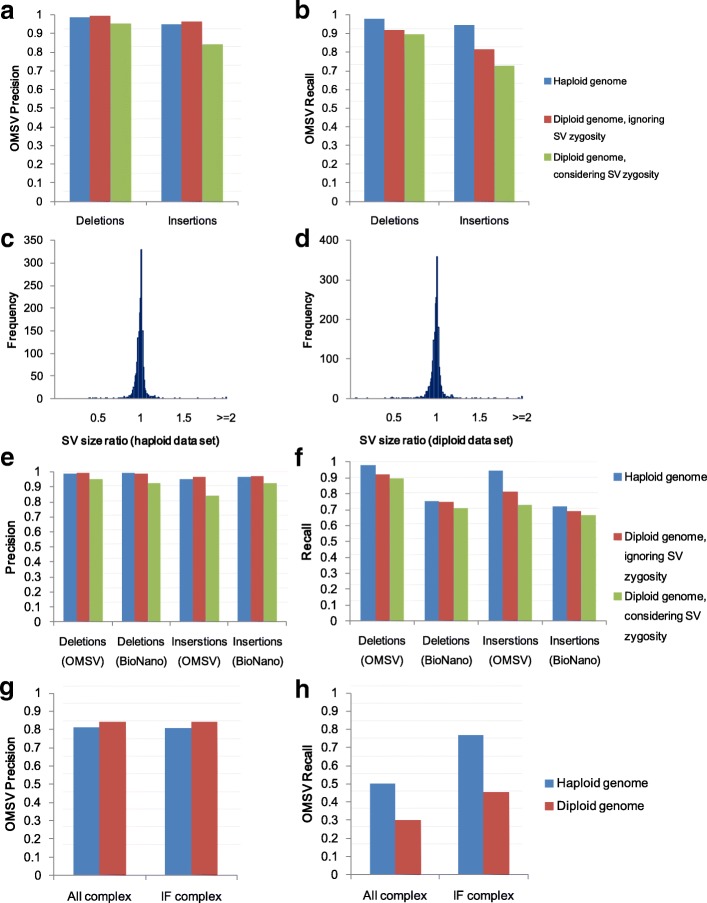
Results based on the default simulated data sets. Precision (**a**) and recall (**b**) of OMSV. Ratio of SV sizes determined by OMSV to their actual sizes, for the haploid (**c**) and diploid (**d**) data sets. Precision (**e**) and recall (**f**) of OMSV compared to BioNano Solve. Results in (**a**) to (**f**) are all based on insertions and deletions larger than 2 kbp. Precision (**g**) and recall (**h**) of OMSV in calling complex SVs from the simulated data, including the whole set (All) and only the intrinsically feasible (IF) ones. IF, intrinsically feasible; SV, structural variation

Here we first focus on insertions and deletions (indels) larger than 2 kbp in the data sets with the default setting, since they constitute a large fraction of our simulated SVs and these large SVs are difficult for short-read-based methods to identify accurately. For the haploid genome, both the precision and recall of OMSV were 98 % for deletions and 95 % for insertions (Fig. 3 a, b), showing that it was highly effective. For the diploid genome, when the goal was to identify SV locations only without considering the correctness of zygosity, OMSV again achieved high precision (99 %) and recall (92 %) for deletions, and high precision (97 %) and moderate recall (81 %) for insertions due to fewer optical maps supporting the SVs in the heterozygous cases. When correct zygosity was also required for an SV to be considered correctly called, OMSV still achieved 90 % precision and 81 % recall on average. For the SVs correctly identified from the two data sets, we further compared their sizes estimated by OMSV with the actual sizes up to the closest defining nicking sites, and found them to be very similar in most cases (Fig. 3 c, d), with a median size ratio of 1.0028 and 1.0029 for the haploid and diploid data sets, respectively.

To benchmark the performance of OMSV, we compared it with the latest version of the assembly-based SV caller BioNano Solve, which is the only other SV caller for nanochannel-based OM with publicly available software (the SV caller used in Cao et al. [15] is a previous version of this software). We found that the precision of the two methods was comparable, but OMSV had 10–31 % higher recall (Fig. 3 e, f). Moreover, since BioNano Solve required a de novo assembly of the optical maps, its running time was 16–21 times longer than OMSV (including the alignment time).

To evaluate the robustness of OMSV, we performed three additional sets of tests. First, using the diploid data set with default settings, we checked the SVs with various numbers of optical maps aligned to their loci and having different likelihood ratios as computed by OMSV. We found that OMSV’s precision remained highly stable at different values of these variables (Additional file 1: Figure S2), and the default parameter values of OMSV (at least ten aligned optical maps and a null-to-alternative likelihood ratio of at most 10^−6^ for an SV to be called) provided a good trade-off between precision and recall. Second, we compared the performance of OMSV on data sets with different depths of coverage. To separate the effects of alignment errors and SV-calling errors, we also considered an idealized situation with no alignment errors (“Methods”). The coverage depth was found to have virtually no effect on the precision of OMSV for the depths considered (Additional file 1: Figure S3a), but it correlated with the recall (Additional file 1: Figure S3b), with almost no SVs being called when the coverage went down to around 5 ×. Importantly, by comparing our results produced with and without optical map alignments, we found that the decreased recall at low coverage depth was largely due to alignment errors, as seen by the big drop in recall with the actual alignment compared to perfect alignment at the same data coverage. Third, we altered the false positive and false negative rates of the OM data, and found that the performance of OMSV remained stable for most settings until the error rates reached unrealistically large values not typically seen in real data (Additional file 1: Figures S4 and S5). Again, we found that the performance drop at high false positive and false negative rates correlated strongly with alignment errors, and thus the performance of OMSV should be automatically improved with better alignment accuracy. Overall, these three sets of tests show that OMSV is generally robust against different data properties and parameter settings.

We also compared different alignment strategies involving alignments from only one of the two aligners, their intersection, and their union. The results (Additional file 1: Figure S6) show that taking the union of the two aligners had the best trade-off between precision and recall, especially when the data set had a low coverage depth.

For complex SVs (Fig. 3 g, h), OMSV achieved 80–85 % precision but only 30–50 % recall on the two default sets. Many of the missed SVs were intrinsically infeasible to call, including inversions that contain no nicking sites or symmetric nicking site patterns that do not change on inversion. After filtering out these cases, the recall rate of the resulting intrinsically feasible (IF) complex SVs was substantially improved to 45–80 %. BioNano Solve contained a function for calling complex SVs, but failed to detect any of them from the simulated data.

Taken together, the simulation results show that OMSV can identify large SVs accurately and comprehensively on data sets with properties typical of real data.

In terms of the running time for OMSV, the main bottleneck was the optical map alignments (Additional file 1: Table S4). This limitation can be overcome by running the aligners on multiple threads in parallel, leading to an overall running time for OMSV of less than 5 h for each simulated human sample with a 100 × genome coverage.

### OMSV identifies SVs concordantly from different members of a family

We next tested OMSV on the optical maps produced from a family trio in a former study [28] (Additional file 1: Table S5). Genetic variants from this trio have been previously reported [29], but they are mostly small variants. OMSV called 1,054–1,126 large indels from the three samples independently (Additional file 1: Table S6, Additional file 2, “Methods”), with an average size of 6.4 kbp and a maximum of 89 kbp. In addition, there were 22 other loci with two indels called at the same locus. OMSV also called 86–158 complex SVs from the three individuals in this trio. Since the actual SVs in these individuals were not known, we used four different methods to estimate the accuracy of OMSV.

First, we hid the sex of the samples from OMSV, and checked the number of SV-calling errors related to the sex chromosomes (Additional file 1: Table S6). When pseudo-autosomal regions were excluded, for the female samples NA12878 and NA12892, 59 and 55 SVs were called on the X chromosome, respectively, whereas no SVs were wrongly called on the Y chromosome. In terms of zygosity, the male sample NA12891 had 18 indels wrongly called as heterozygous among the 53 indels called on the non-pseudo-autosomal regions of the sex chromosomes. Based on these numbers, the estimated zygosity precision was (53−18)/53=66 %.

Second, we compared the indels called from the three individuals. Among the high-confidence calls (“Methods”), 99 % were concordant with Mendelian inheritance when the zygosity of the SVs was ignored, and 86 % were concordant when the zygosity was considered. We used the precision and recall from our simulations to estimate the expected Mendelian concordance to be 96 % when zygosity was ignored and 83 % when zygosity was considered (“Methods”), suggesting that the accuracy of OMSV on the trio data was comparable to that on the simulated data.

Third, we compared our SV calls with the manual checks made by Mak et al. [28] based on nicking site patterns of aligned molecules (Additional file 1: Table S7). Among our SVs with manual checking results for the three individuals, 96–97 % of them were considered correct by the manual checking results when zygosity was ignored, which is similar to the precision values in the simulation study. When zygosity was considered, 73–74 % of our SVs were considered correct by the manual checking results, which is lower than that in the simulation. Together, these results suggest that OMSV could identify SV locations accurately but determining the correct zygosity of SVs could be more difficult with real data.

Finally, we compared our indel list for NA12878 with two lists of indels previously detected from this sample using sequencing-based methods [2, 30]. Focusing on large (>2 kbp) indels, the intersection of the OMSV list and either of these two sequencing-based lists (81 and 90 indels, respectively) was similar to the intersection of these two lists (84 indels) (Additional file 1: Figure S7). Interestingly, 500 (96 %) of the insertions and 178 (38 %) of the deletions called by OMSV were unique among the three lists. Based on the above estimation of the accuracy of OMSV, a large fraction of these novel indels are expected to be real. These observations suggest that OMSV is able to identify SVs commonly called by other sequencing-based methods as well as uncover novel ones missed by them.

**Fig. 4:**
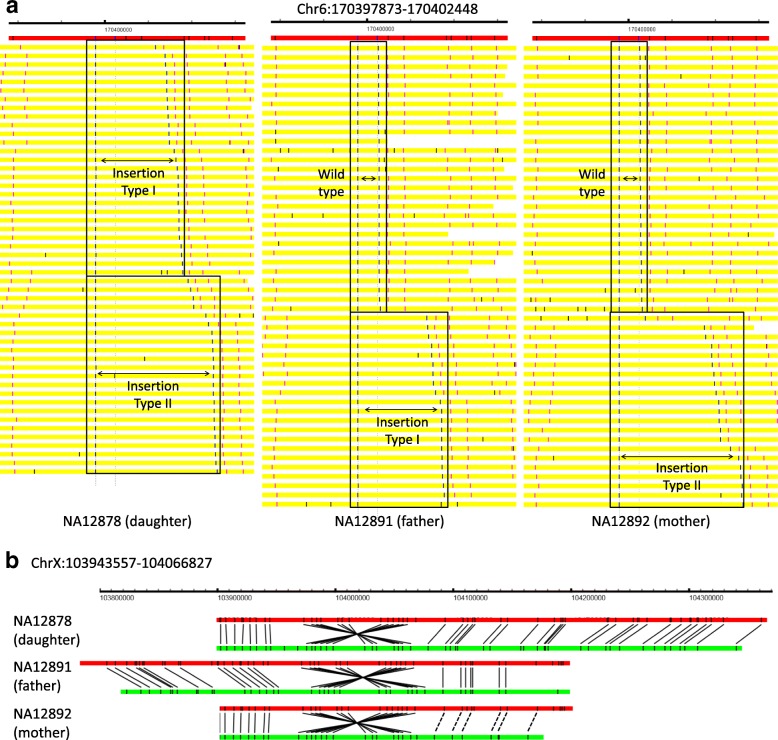
Examples of SVs identified from the trio. **a** An insertion identified on chromosome 6, visualized by OMView [51] using the anchor view with the nicking site immediately before the insertion as the anchor. The red horizontal bars show the reference, with the nicking sites marked as black vertical lines. Each yellow horizontal bar represents an optical map, with the two aligned nicking site labels defining the SVs in blue, other aligned labels in pink, and unaligned labels in black. For each individual, optical maps are arranged into different sections based on the allele that they support. The father has a heterozygous insertion of around 14.6 kbp (insertion type I) and the mother has a heterozygous insertion of around 22.7 kbp (insertion yype II). The daughter inherited both insertions from her parents. **b** An inversion identified on chromosome X, visualized using the alignment view of OMView. For each individual, the top horizontal bar shows the reference and the bottom horizontal bar shows a representative optical map. Black solid and dashed lines linking the reference and the optical map, respectively, represent aligned nicking sites and nicking sites that should probably be aligned but were missed by the alignment pipeline

We select two examples to illustrate the SVs identified by OMSV. In the first example for chromosome 6 (Fig. 4 a), the father (NA12891) has a heterozygous insertion of around 14.6 kbp, the mother (NA12892) has a heterozygous insertion of around 22.7 kbp, and the daughter (NA12878) has inherited both insertions from the parents. This example demonstrates the capability of OMSV to identify heterozygous SVs and loci with two distinct alleles both different from the reference. In the second example (Fig. 4 b), a large inversion of around 123.3 kbp was consistently found on chromosome X from all three individuals, with clear nicking site patterns that support the inversion.

### OMSV identifies many SVs missed by short-read-based SV callers

To evaluate further the capability of OMSV to detect novel SVs, we produced optical maps from the human C666-1 cell line [31] (Additional file 1: Table S8). C666-1 cells consistently harbor multiple Epstein–Barr virus (EBV) episomes. As a first check of the data produced, we aligned the optical maps to the EBV reference in C666-1 [32], and found a large number of well-aligned optical maps (Additional file 1: Figure S8). Comparing the average coverage depth of the optical maps aligned to the human (72 ×) and EBV (847 ×) references, we estimated an average of 24 copies of the EBV genome per C666-1 cell, which is highly consistent with a previous estimate based on sequencing data [33].

We then applied OMSV to identify SVs in the C666-1 cellular genome (Additional file 1: Table S9, Additional file 3). In total, 810 loci containing indels larger than 2 kbp were called, with an average size of 6.6 kbp and a maximum of 106 kbp. Among the large indels identified, 67 % were insertions while 33 % were deletions, and 69 % were homozygous while 31 % were heterozygous. Since C666-1 was originally derived from a male sample, we checked the number of indels wrongly called as heterozygous on the sex chromosomes (Additional file 1: Table S9), and found six such errors among the 21 (29 %) SVs identified, which is close to the error rate we obtained from NA12891 (34 %). To investigate the origin of our identified indels, we intersected them with segmental duplications in the human genome [34, 35]. We found 143 of the C666-1 large indels overlapping with segmental duplication regions, among which 78 involved segmental duplications that overlap exons of protein-coding genes (Additional file 4). Therefore, at least 18 % of the SVs found in C666-1 were likely due to common segmental duplications, while others could be more specific to C666-1.

**Fig. 5:**
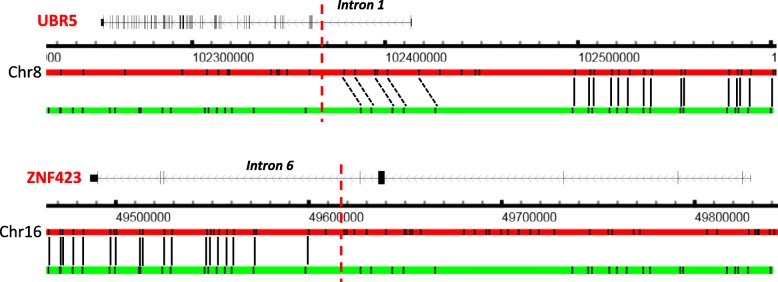
The previously reported UBR5–ZNF423 translocation in C666-1 re-identified by OMSV, visualized using the alignment view of OMView. For each of the two gene loci, the top horizontal bar shows the reference and the bottom horizontal bar shows a representative optical map. Black solid and dashed lines linking the reference and the optical map represent, respectively, aligned nicking sites and nicking sites that should probably be aligned but were missed by the alignment pipeline. The vertical red dashed lines show the break points previously reported [36]

In addition to indels, OMSV also identified 68 copy number variations (CNVs), 28 medium-sized inversions, 13 large inversions, and six translocations (two intra-chromosomal and four inter-chromosomal) (Additional files 3 and 5). A translocation in C666-1 between intron 1 of UBR5 and intron 6 of ZNF423 was previously reported, leading to a fusion transcript [36]. We were able to confirm the existence of this translocation in the list of complex SVs identified by OMSV (Fig. 5).

**Fig. 6:**
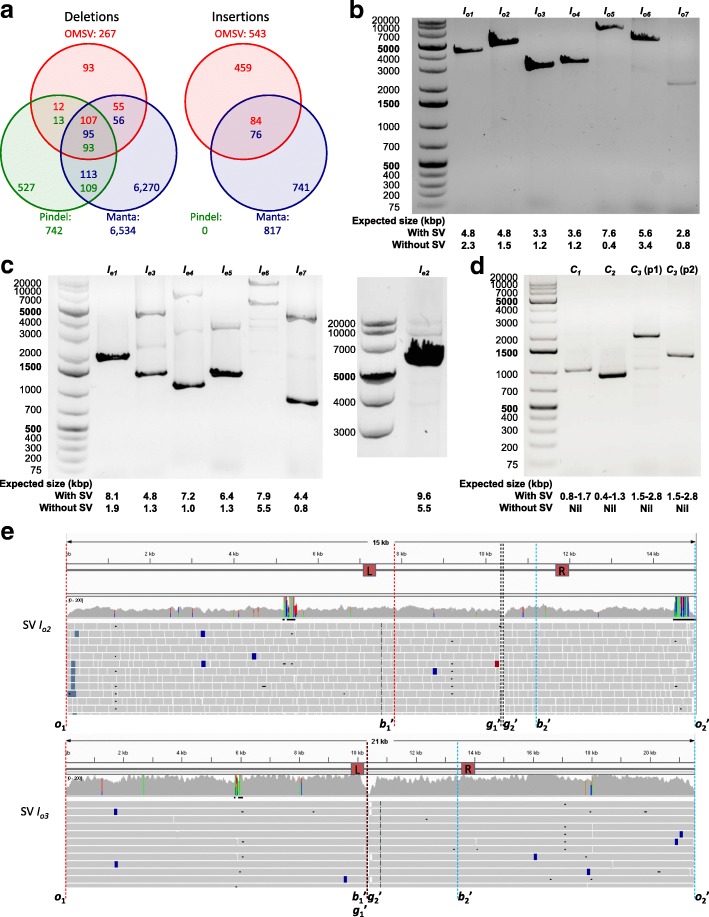
SVs identified by OMSV from C666-1. **a** Overlap between the large (>2 kbp) indels identified by OMSV and the two short-read-based callers, Manta and Pindel. In the common regions, the number of a certain color indicates the number of SVs called by the respective method that overlap SVs called by the other method(s). **b–d** Polymerase chain reaction results for selected homozygous insertions (**b**), heterozygous insertions (**c**), and complex SVs (**d**). For the heterozygous insertions, *I*_*e*2_ was tested separately from the other six cases due to the large expected product size of its insertion allele. For the inversion case *C*_3_, p1 and p2 correspond to the two primer pairs. **e** Alignment of sequencing reads to the inferred C666-1 sequences of SV *I*_*o*2_ and SV *I*_*o*3_. The L and R boxes mark the primer locations. Definitions of *o*_1_, *o*2′, *b*1′, *b*2′, *g*1′ and *g*2′ are given in Additional file 1: Figure S9. Sequencing read alignments are visualized by IGV [49]. SV, structural variation

Whole-genome sequencing data for C666-1 were previously produced at 75 × coverage with 100 bp paired-end reads and an average insert size of 290 bp [32]. We used two sequencing-based SV callers, Manta [37] and Pindel [38], to identify large (>2 kbp) SVs from the sequencing data. Among the 810 indels identified by OMSV, 552 of them (68 %) were missed by both short-read-based SV callers (Fig. 6 a). In particular, among the 543 insertions, 459 of them (85 %) were missed by both. Even for the insertions detected by Manta or Pindel, only the locations of the break points were provided, but not the sizes of the insertions, which are reported by OMSV.

On the other hand, some large SVs were called by the short-read-based methods but not by OMSV. Assuming that SVs identified by at least two methods are more likely to be real, we found that OMSV had the highest fraction of deletions belonging to this category of high-confidence SVs (174/267=0.65), compared to Manta (264/6534=0.04) and Pindel (215/742=0.29). Since Pindel did not call any large insertions, we could not perform this analysis on the insertions.

We further investigated the 111 non-redundant deletions commonly called by Manta and Pindel but not by OMSV. We found that two overlapped with N-gaps in the reference genome or fragile sites and 34 were in regions with low optical map coverage, both representing SVs that are impossible for OMSV to detect based on the data produced. Another 18 cases were missed by OMSV due to errors in the alignment of optical maps. There were 33 cases in which the alignments of the optical maps were good but did not support an SV, which could mean either the optical maps supporting the SVs were not aligned successfully or the SVs identified by the short-read-based callers were false positives. Of the remaining cases, 19 had low likelihood scores that could not pass the OMSV parameter threshold we chose, and five were missed by OMSV for no obvious reasons. The 24 SVs in these last two categories may be detectable by improving the SV-calling modules in OMSV.

Of the 115 complex SVs identified by OMSV, Manta and Pindel together detected only one large inversion and two translocations. On the other hand, these two short-read-based methods identified only eight inversions in common, with none of the 116 translocations detected by Manta also detected by Pindel.

To check further the accuracy of the SVs identified by OMSV, we performed a polymerase chain reaction (PCR) validation. We focused on insertions and complex SVs, which are more difficult for short-read-based callers to identify accurately. Considering the maximum possible product size of PCR, we selected 17 SVs for validation experiments, including seven homozygous insertions, seven heterozygous insertions, and three complex SVs (Additional file 1: Tables S10–S12). For each of these, we designed primers based on its predicted break points on the reference sequence, and compared the length of the resulting PCR-amplified product with its expected length with or without the SV (Additional file 1: Tables S10–S13, “Methods”).

For the homozygous insertions (Fig. 6 b), all seven cases showed a single band much closer to the expected size with the insertion than the expected size without the insertion, although in one case (*I*_*o*7_), the band was weak.

For the heterozygous insertions (Fig. 6 c), the two bands having the expected product sizes with or without the insertions were seen in four of the seven cases (*I*_*e*2_– *I*_*e*4_ and *I*_*e*7_), although the one corresponding to the insertion allele was weaker in general, likely due to their longer products. Additional bands were also observed in several cases, suggesting that the insertions could be due to tandem duplications and the additional bands correspond to another copy number. For two of the cases (*I*_*e*5_ and *I*_*e*6_), a band was observed at the expected size of the reference allele, while another relatively strong band was observed with a size slightly different from the expected size with the insertion, illustrating a limitation of precisely estimating SV sizes from optical maps. Finally, for one case (*I*_*e*1_), only one band was observed at the expected size of the reference allele, indicating that it could be a false positive call.

For the complex SVs (Fig. 6 d), in all three cases, PCR products were seen with a size in agreement with the size estimated by OMSV.

Altogether, among the 17 validated cases, 14 were clearly validated, two had issues with the estimated SV size, and one could not be validated.

Since optical maps estimate SV break points only up to the closest nicking sites, we used the sequencing reads to determine the break points more precisely and to deduce the inserted sequences in insertions using local sequence assembly (Additional file 1: Figure S9, “Methods”). The inferred sequences for the seven PCR-validated homozygous insertions and the precise SV break points are all supported by a large number of aligned sequencing reads (Fig. 6 e, Additional file 1: Figure S10).

## Discussion

Currently, it is difficult to detect large or complex SVs from sequencing alone, and even harder to estimate SV sizes, due to the short read length and limited insert size between read pairs. In particular, large insertions are especially difficult for short-read-based SV-calling methods to detect since the alignment of supporting reads that contain contents not in the reference is difficult, and the read coverage is only locally dropped around the insertion site. Having repeat elements around the SV break points could also make SV detection from short sequencing reads difficult. In contrast, using nanochannel-based optical maps, whole SVs are easily contained in a single optical map, making SV detection highly feasible and accurate. Here we demonstrated that OMSV is a powerful tool for identifying large SVs, ranging from kilobases to more than a hundred kilobases. In fact, as long as an optical map can be correctly aligned to the reference by having sufficient nicking sites in the flanking non-SV portions, the larger an SV is, the easier it is for OMSV to detect it, since the corresponding distance change between the defining nicking sites is less likely due to scaling and measurement errors alone. This property makes OMSV an ideal complement to sequencing-based SV callers, which are generally more accurate in detecting smaller SVs.

OMSV detects complex regions and very large SVs with a two-round alignment strategy that allows split-alignment of an optical map to multiple locations on the genome. Split-alignments of optical maps could come with a cost of extra alignment time. One way to tackle this problem is first to quickly align optical maps that can be aligned to single genomic loci using a standard aligner, and then apply the split-alignment strategy only to the remaining unaligned optical maps.

Since the SV-calling modules require only a list of optical map alignments as input, the alignment methods used in the OMSV pipeline can be flexibly changed to other choices. Besides, if a high-quality de novo assembly of the optical maps is available, the optical maps can also be first aligned to the assembly, and their alignment to the reference can then be inferred from further aligning the assembly to the reference. For optical maps that deviate significantly from the reference map, this two-step alignment strategy could be more accurate than directly aligning optical maps to the reference.

With each optical map coming from one DNA molecule, OMSV can potentially be extended to study haplotypes, cell-type composition in a sample, and cell-to-cell variability. These analyses would require highly accurate alignments of individual labels of the optical maps. Probing the nicking sites of a second enzyme using an additional color channel may further improve the alignment accuracy necessary for these analyses. With such improved accuracy, we also hope to extend OMSV to call the zygosity of complex SVs.

## Conclusions

In this paper, we described the OMSV pipeline for identifying SVs from nanochannel-based optical maps. The accuracy of OMSV has been confirmed by both simulations and optical maps from a family trio. OMSV outperformed the only publicly available tool for SV detection from OM data in three aspects: (1) OMSV identified many more SVs at a precision level similar to that of this method, (2) OMSV identified many of the complex SVs but this method missed all of them, and (3) OMSV ran much faster as it does not require a time-consuming de novo assembly of the optical maps.

We also used OMSV to identify SVs from the C666-1 cell line, and found 68 % of them were missed by sequencing-based SV callers, including 85 % of the insertions. Some of these SVs were experimentally validated independently.

We provide OMSV as open-source software, which can be used routinely in genome projects to identify large SVs accurately and comprehensively. This will likely have important implications for understanding genetic diversity and disease susceptibility.

## Methods

### A complete error model for optical maps

We modeled the generation of optical maps from a DNA sequence as a random process with various types of error. This combines some ideas previously proposed [19, 21] and several new components based on properties observed in real human optical maps [28].

In our model, the starting locations of *n* DNA fragment molecules are first uniformly and independently sampled from the DNA sequence. Each of these starting locations is used to produce a molecule of length *l*_0_+*l*_*v*_, where *l*_0_ is a constant minimum molecule length and *l*_*v*_ is a random variable that follows a Poisson distribution with mean *μ*_*l*_. In real experiments, *l*_0_ is a threshold chosen such that molecules shorter than it are excluded from the analyses.

The restriction sites on each molecule can be identified by matching its sequence against the recognition motif of the nicking enzyme selected. In our model, each restriction site has a false negative rate of *f*_−_ for not having a corresponding observable label in the optical map due to incomplete enzymatic digestion or a measurement error.

False positive labels not originating from actual restriction sites but caused by artifacts such as non-specific enzymatic cuts are then introduced. For every two adjacent restriction sites, the number of false positive labels is randomly sampled from a Poisson distribution with mean *df*_+_, where *d* is the distance between the two sites and *f*_+_ is the false positive rate. If the resulting number of false positive labels is non-zero, the locations of these false positives are uniformly and independently sampled from the locations between the two sites.

After these steps, each random molecule is represented by a list of distances between adjacent observed labels (including both true positives and false positives). For the convenience of discussion, we also assume the beginning and end of each molecule are marked by two artificial labels, the locations of which in actual optical maps can be determined by the span of the stained DNA backbone. Each molecule then undergoes a random stretch or compression to model sizing errors in the experiments, by multiplying the distance between every two observed labels by a factor *α*, where *α* is sampled from a Cauchy distribution with the values of the location and scale parameters set to *o*_*α*_ and *s*_*α*_, respectively. We chose the Cauchy distribution since it had a good fit with the real data we produced (Additional file 1: Figure S11).

To model the finite resolution of optical measurements, any two adjacent labels on a stretched/compressed molecule at a distance of *d* bp from each other are merged into one single label at their midpoint with a probability of

$$1 - \frac{1}{1 + \exp\left[-0.01(d-d_{1/2})\right]}, $$

where *d*_1/2_ is a reference distance at which the chance for the two labels to be merged is 1/2.

Finally, measurement errors are modeled by moving each label by an offset that follows a uniform distribution defined on [−*e, e*] for a given parameter *e*.

### SV-calling modules

Based on the above generative model, we developed two statistical modules for identifying SVs from optical maps. The first module looks for individual extra or missing sites on the molecules compared to the reference sequence. Some small SVs with only a small change in the distance between restriction sites are detected better by this method. The second module compares the distance between two restriction sites on the molecules with that on the reference genome. It can detect larger SVs not necessarily involving extra or missing restriction sites.

Both modules require an alignment of the optical maps to a reference map obtained from the in silico digestion of the reference sequence, where adjacent labels are merged in the way described above. Based on the alignments, OMSV extracts three types of information as input to the two SV-calling modules: (1) the expected locations of restriction sites on the reference sequence, (2) the distance (in base pairs) between every two adjacent observed labels on each molecule, and (3) an alignment of the labels on the molecules to the restriction sites on the reference. Every label can be aligned to zero or one restriction site on the reference, and each restriction site on the reference can be aligned to zero or one label on each molecule.

There is a third module, which uses additional alignment and coverage information to identify complex SVs.

#### Module for identifying SVs involving extra or missing restriction sites

To identify missing restriction sites on the molecules, we adopted a method originally developed for refining optical map assemblies [20], and extended it to detect both homozygous and heterozygous genetic variants.

Suppose there are *M* molecules aligned to a region that covers a restriction site on the reference sequence, of which *m* support the existence of the restriction site (Fig. 2 a). Each of the *m* supporting molecules either actually contains the site or has a false positive label. Each of the *M*−*m* non-supporting molecules either actually does not contain the site or has a false negative. We consider three hypotheses for the observed data: (1) the null hypothesis $H_{0}^{(\text {miss})}$ that the restriction site actually exists on the subject DNA sequence in homozygous form (and thus, there are no false positives), (2) the first alternative hypothesis $H_{\text {hom}}^{(\text {miss})}$ that the site is missing on the subject sequence in homozygous form (and thus, there are no false negatives), and (3) the second alternative hypothesis $H_{\text {het}}^{(\text {miss})}$ that the site is missing on the subject sequence in heterozygous form.

Under the null hypothesis $H_{0}^{(\text {miss})}$, the probability of observing *m* or fewer supporting molecules is

$$\Pr\left(x \leq m | H_{0}^{(\text{miss})}\right) = \sum\limits_{x=0}^{m} \left(\underset{x}{M}\right) {(1 - \text{fn})}^{x} {\text{fn}}^{M-x}, $$

where fn is the false negative rate to be estimated from the observed data. Similarly, depending on whether $H_{0}^{(\text {miss})}$, $H_{\text {hom}}^{(\text {miss})}$ or $H_{\text {het}}^{(\text {miss})}$ is true, the data likelihood is, respectively,

$$L_{H_{0}^{(\text{miss})}} = \left(\underset{m}{M}\right) {(1 - \text{fn})}^{m} {\text{fn}}^{M-m}, $$

$$L_{H_{\text{hom}}^{(\text{miss})}} = \left(\underset{m}{M}\right) {\text{fp}}^{m} {(1 - \text{fp})}^{M-m}, \text{and}$$

$$\begin{array}{*{20}l} L_{H_{\text{het}}^{(\text{miss})}} &= {\sum\nolimits}_{k=0}^{M} \left\{\left(\underset{k}{M}\right) \left(\frac{1}{2}\right)^{M} \sum\limits_{l=\max(0, m-M+k)}^{\min(k,m)}\right.\\ &\qquad\qquad\quad\left[\left(\underset{l}{k}\right) {(1 - \text{fn})}^{l} {\text{fn}}^{k-l} \left(\underset{m-l}{M-k} \right)\right.\\&\qquad\qquad\qquad\left.\left.{\text{fp}}^{m-l} {(1 - \text{fp})}^{M-k-m+l} {\vphantom{\left(\underset{l}{k}\right)}}\right]{\vphantom{0\sum\limits^{a}_a}}\right\}, \end{array} $$

where fp is the false positive rate to be estimated from the observed data, *k* is, in the heterozygous case, the unknown number of molecules from the chromosome with the restriction site, and *l* is the number of molecules among the *k* on which the restriction site is observed. In the model, we assume for a molecule that there is an equal probability of it coming from either chromosome.

Based on these definitions, if both the *p* value $\Pr \left (x \leq m | H_{0}^{(\text {miss})}\right)$ and the likelihood ratio $L_{H_{0}^{(\text {miss})}} / \max \left (L_{H_{\text {hom}}^{(\text {miss})}},L_{H_{\text {het}}^{(\text {miss})}}\right)$ are smaller than the corresponding thresholds for a site, it is considered a homozygous missing site if $L_{H_{\text {hom}}^{(\text {miss})}} \geq L_{H_{\text {het}}^{(\text {miss})}}$ or a heterozygous missing site if $L_{H_{\text {hom}}^{(\text {miss})}} < L_{H_{\text {het}}^{(\text {miss})}}$.

A similar procedure is used for calling homozygous and heterozygous extra restriction sites. Suppose there are *M* molecules aligned to a region on the reference sequence, among which *m* support the existence of a restriction site in a region that does not exist according to the reference sequence. Under the null hypothesis $H_{0}^{(\text {extra})}$ that the site is absent in homozygous form, the probability of observing *m* or more supporting molecules is

$$\Pr\left(x \geq m | H_{0}^{(\text{extra})}\right) = \sum\limits_{x=m}^{M} \left(\underset{x}{M}\right) {\text{fp}}^{x} {(1 - \text{fp})}^{M-x}. $$

Similarly, depending on whether the site is absent in homozygous form $\left (\text {null hypothesis} H_{0}^{(\text {extra})}\right)$, exists in homozygous form $\left (\text {alternative hypothesis} H_{\text {hom}}^{(\text {extra})}\right)$, or exists in heterozygous form $\left (\text {alternative hypothesis} H_{\text {het}}^{(\text {extra})}\right)$, the data likelihood is defined as

$$L_{H_{0}^{(\text{extra})}} = L_{H_{\text{hom}}^{(\text{miss})}}, $$

$$L_{H_{\text{hom}}^{(\text{extra})}} = L_{H_{0}^{(\text{miss})}}, $$

and

$$L_{H_{\text{het}}^{(\text{extra})}} = L_{H_{\text{het}}^{(\text{miss})}}. $$

Based on these definitions, if both the *p* value $\Pr \left (x \geq m | H_{0}^{(\text {extra})}\right)$ and the likelihood ratio $L_{H_{0}^{(\text {extra})}}/\max \left (L_{H_{\text {hom}}^{(\text {extra})}},L_{H_{\text {het}}^{(\text {extra})}}\right)$ are smaller than the corresponding thresholds for a site, it is considered a homozygous extra site if $L_{H_{\text {hom}}^{(\text {extra})}} \geq L_{H_{\text {het}}^{(\text {extra})}}$ or a heterozygous extra site if $L_{H_{\text {hom}}^{(\text {extra})}} < L_{H_{\text {het}}^{(\text {extra})}}$.

In practice, we also define a minimum number of supporting molecules *M*_min_. For any site with less than *M*_min_ molecules covering the locus (regardless of whether they support the presence of the restriction site or not), we did not call genetic variants from it since the result would not be reliable.

#### Module for identifying SVs involving large size changes

Large SVs are usually associated with a deviation of the distance between two restriction sites on the reference sequence (Fig. 2 b, *d*_0_) and that on the molecules (*d*_1_), which may or may not involve extra or missing restriction sites on the molecules. To identify these cases systematically, we first check the distances between every two adjacent restriction sites on the reference sequence and compare them with the corresponding label distances on the aligned molecules (which would cover the first two cases of Fig. 2 b). We then check the distances between every two adjacent labels on the aligned molecules that have not been checked, and compare them with the distance between the aligned restriction sites on the reference (which would cover the third case). Each of these checks is performed with the following statistical method.

Suppose there are two (not necessarily adjacent) restriction sites on the reference sequence with a distance *d*_0_, and there are *M* aligned molecules covering the region. Suppose the distances of the corresponding aligned labels on the molecules are *d*_1_,*d*_2_,…,*d*_*M*_, where *d*_1_≤*d*_2_≤⋯≤*d*_*M*_. Our method computes the ratios *r*_*i*_=*d*_*i*_/*d*_0_ for each of the *M* molecules. It then compares the following hypotheses according to the error model we defined:

1. Null hypothesis *H*_0_, that there are no insertions or deletions between the two sites
2. *H*_hom_, that there is a homozygous indel between the two sites
3. $H_{\text {het}}^{(\text {ins})}$, that there is a heterozygous insertion between the two sites
4. $H_{\text {het}}^{(\text {del})}$, that there is a heterozygous deletion between the two sites
5. *H*_tri_, that the locus is triallelic, i.e., there are two different insertions, two different deletions, or one insertion and one deletion between the two sites, where each chromosome bears one of the two variant alleles

Under the null hypothesis *H*_0_, the likelihood of observing the distance ratios *r*_1_,*r*_2_,…,*r*_*M*_ is

$$L_{H_{0}} = \prod_{i=1}^{M} {Cauchy}(r_{i}, r_{0}, \gamma), $$

where *Cauchy*(*r*_*i*_,*r*_0_,*γ*)=*γ*/(*π*[(*r*_*i*_−*r*_0_)^2^+*γ* ^2^]) is the probability density function of the Cauchy distribution with position parameter *r*_0_ and scale parameter *γ*. In SV detection, using the Cauchy distribution to model the distance ratios has an advantage that it is not heavily affected by extreme outliers caused by alignment errors.

Under the alternative hypothesis *H*_hom_, the distance ratios *r*_1_,*r*_2_,…,*r*_*M*_ are sampled from a Cauchy distribution with a different value for the location parameter but the same value *γ* for the scale parameter. The likelihood of observing the distance ratios is, therefore,

$$L_{H_{\text{hom}}} = \prod_{i=1}^{M} {Cauchy}(r_{i}, r_{0}{\prime}, \gamma), $$

where *r*0′ is the location parameter of the distribution of distance ratios for this indel event. Finding the maximum likelihood estimate of *r*0′ would require using numerical methods to solve a high-degree polynomial. Instead, we used the sample median of the *M* *r*_*i*_’s as an imperfect estimate [39].

Under the alternative hypothesis $H_{\text {het}}^{(\text {ins})}$, some of the distance ratios are sampled from the null distribution and the others are sampled from an alternative Cauchy distribution with a larger value *r*_0_′ for the location parameter but the same value for the scale parameter. The likelihood of the distance ratios is $ L_{H_{\text {het}}^{(\text {ins})}} = \frac {1}{2^{M}} {\sum \nolimits }_{S\subset \{1,2,\dots,M\}} \left [\prod _{j\notin S}{Cauchy}(r_{j}, r_{0}, \gamma) \prod _{i\in S}\right.\left.{Cauchy}(r_{i}, r_{0}{\prime }, \gamma)\right ], $ where *S* represents the set of molecules from the chromosome with the insertion, assuming there is an equal probability for each molecule coming from either chromosome. Practically, this likelihood is difficult to compute due to the exponential number of terms in the summation. We made an assumption that the two distributions are sufficiently separated, with |*r*_0_′−*r*_0_|≫*γ*. Based on this assumption, we consider only the summation terms of which *S* takes the form {*r*_*M*−*k*+1_,*r*_*M*−*k*+2_,…,*r*_*M*_}, which involves only the *k* largest distance ratios. We then try all possible values of *k* such that at least *k*_min_ molecules come from each chromosome (Fig. 2 c). As a result, the likelihood formula is simplified as $ L_{H_{\text {het}}^{(\text {ins})}} = \frac {1}{2^{M}} {\sum \nolimits }_{k=k_{\text {min}}}^{M-k_{\text {min}}} \left [\prod _{i=1}^{M-k} {Cauchy}(r_{i}, r_{0}, \gamma) \prod _{j=M-k+1}^{M}\right.\left.{Cauchy}(r_{j}, \tilde {\mu }_{M-k+1..M}, \gamma) \right ], $ where $\tilde {\mu }_{M-k+1..M}$ is the sample median of *r*_*M*−*k*+1_, *r*_*M*−*k*+2_, …, *r*_*M*_.

Similarly, for heterozygous deletions, a simplified likelihood formula is defined as $ L_{H_{\text {het}}^{(\text {del})}} = \frac {1}{2^{M}} \sum _{k=k_{\text {min}}}^{M-k_{\text {min}}} \left [\!\prod _{i=1}^{k} \!{Cauchy}(r_{i}, \tilde {\mu }_{1..k}, \!\gamma \!) \prod _{j=k+1}^{M} {Cauchy}(r_{j}, r_{0}, \gamma)\right ], $ where $\tilde {\mu }_{1..k}$, the sample median of *r*_1_,*r*_2_,…,*r*_*k*_, is expected to be smaller than *r*_0_ in this case (and a heterozygous deletion would not be called if this expectation is not satisfied).

For the triallelic cases, the simplified likelihood formula is defined as $ L_{H_{\text {tri}}} = \frac {1}{2^{M}} {\sum \nolimits }_{k=k_{\text {min}}}^{M-k_{\text {min}}}\left [\prod _{i=1}^{k} {Cauchy}(r_{i}, \tilde {\mu }_{1..k}, \gamma) \prod _{j=k+1}^{M} {Cauchy}(r_{j}, \tilde {\mu }_{k+1..M}, \gamma)\right ]\!, $ where $\tilde {\mu }_{1..k}$ is the median of *r*_1_,*r*_2_,…,*r*_*k*_ and $\tilde {\mu }_{k+1..M}$ is the median of *r*_*k*+1_,*r*_*k*+2_,…,*r*_*M*_.

Finally, our method compares the likelihood values. If the likelihood ratio $ \frac {L_{H_{0}}}{\max \left \{L_{H_{\text {hom}}}, L_{H_{\text {het}}^{(\text {ins})}}, L_{H_{\text {het}}^{(\text {del})}}, L_{H_{\text {tri}}}\right \}} $ is smaller than a threshold, an SV is called according to the following rules. If $ \max \left \{L_{H_{\text {hom}}}, L_{H_{\text {het}}^{(\text {ins})}}, L_{H_{\text {het}}^{(\text {del})}}, L_{H_{\text {tri}}}\right \} $ is equal to

- $L_{H_{\text {hom}}}$: If *r*_0_′>*r*_0_, a homozygous insertion is called. Otherwise, a homozygous deletion is called.
- $L_{H_{\text {het}}^{(\text {ins})}}$: A heterozygous insertion is called.
- $L_{H_{\text {het}}^{(\text {del})}}$: A heterozygous deletion is called.
- $L_{H_{\text {tri}}}$: An SV of the multiple type is called. If $\tilde {\mu }_{1..k}$ and $\tilde {\mu }_{k+1..M}$ are both smaller than *r*_0_, two different deletions are called. If both are larger than *r*_0_, two different insertions are called. Otherwise, an insertion and a deletion are called.

Practically, if the distance change is too small, either absolutely or relative to the distance on the reference, the SV calls are less reliable. We, therefore, keep only SVs with a distance change larger than a threshold *δ*, where the distance on the molecules is defined as the median distance of the set of molecules that lead to a term with the largest value in the likelihood calculation.

#### Module for identifying complex SVs

We also developed a module for identifying three types of complex SVs, namely inversions, translocations, and CNVs.

**Using split-alignment to identify large inversions and translocations** The split-alignment capability of OMBlast [22] allows different parts of a single optical map to be separately aligned to different locations of the same chromosome (Fig. 2 d). The default setting of OMBlast limits the maximum distance between these different locations to reduce the false alignment rate and thus, it permits direct calling of only intra-chromosomal translocations involving close loci. To detect other intra-chromosomal translocations and inter-chromosomal translocations, we used a two-round alignment strategy (Fig. 2 e), in which the first round performed standard alignments of optical maps, with some optical maps only partially aligned. For these optical maps, the unaligned regions were then independently aligned again in the second round, thus allowing the detection of all types of translocations. In addition, by allowing different portions of the same optical map to be aligned in different orientations, large inversions can also be detected. To reduce false positives, only translocations and large inversions supported by two or more optical maps are considered.

**Using reverse palindromic CIGAR strings to identify medium-sized inversions** Inversions with a size between 2 and 100 kbp can be contained in a single optical map, and are detected by locating a region in an optical map alignment with (1) a reverse-palindromic CIGAR (Compact Idiosyncratic Gapped Alignment Report) string and (2) matching distances between adjacent restriction sites on the reference and those between adjacent labels on the reversed optical map (Fig. 2 f). In a CIGAR string, a matched, missing, or extra label is denoted as M, D, or I, respectively. The reverse complement of a CIGAR string is its reverse with I’s and D’s interchanged. For example, the reverse complement of MDDI is DIIM. A CIGAR string is reverse palindromic if it is the same as its reverse complement, such as DIDIDI. Two distances *d*_1_ and *d*_1_′ are considered matched if *d*_1_×(1−*e*_*t*_)−*e*_*m*_≤*d*_1_′≤*d*_1_×(1+*e*_*t*_)+*e*_*m*_, where *e*_*t*_ and *e*_*m*_ are the maximum scaling and measurement errors (set to 0.1 and 500 bp), respectively. To control the quality, we called an inversion only if it had at least ten supporting molecules and at least four nicking sites within the inverted region.

**Using coverage depth to identify CNVs** We modified an event-wise significance testing method [40] to identify large CNVs. The original method uses a sliding window (with 100 bp) to scan the reference and look for windows with a coverage depth significantly different from other windows, based on the distribution of depths of windows with similar GC contents. Neighboring windows are then grouped into blocks to identify the span of the CNVs, with a method for correcting for multiple hypothesis testing. To adopt this method for OM data, first the window size was enlarged to 2*d*_1/2_ to accommodate for the lower resolution of OM data, where *d*_1/2_ is the imaging resolution. Then, to determine the statistical significance of each window, instead of grouping windows by GC content, we grouped them by nicking site counts. The depths (number of aligned optical maps) of all windows within a group were fitted to a Gaussian distribution, and a window was considered a CNV candidate if it received a Z-test *p* value <0.05. The same procedure for determining CNV spans in the original method was then applied.

### The overall OMSV pipeline

The overall OMSV pipeline is illustrated in Fig. 1 b. In the alignment pipeline, we used default parameter values for RefAligner and OMBlast for all the simulated and real data except C666-1, for which we used the RefAligner parameter values for complex genomes (available on our supplementary website) suggested by the BioNano technical team. The reference map was deduced from the human reference hg38 in all cases. RefAligner and OMBlast alignments were integrated based on the following rules:

1. If the two methods align an optical map to genomic regions within half the length of the optical map from each other, they are considered to agree on the alignment, and the alignment of RefAligner is taken.
2. If only one of the two methods can align an optical map, the alignment is taken directly.
3. If neither method can align an optical map, or both of them can align but their alignments do not agree with each other, the optical map is left unaligned.

We call this the union strategy in Additional file 1: Figure S6. We also considered an intersection strategy, which involved only the alignments satisfying the first rule above.

The resulting integrated list of alignments is sent to the three modules for SV identification. The results from the three modules are then integrated to form a final list of SVs. The parameter values for OMSV used in our experiments are listed in Additional file 1: Table S14.

### Filtering of SVs detected from real data

We considered only optical map alignments with a confidence score of 9 or more. For the indels identified from the family trio and the C666-1 cell line, we filtered those that overlapped N-gaps, fragile sites, or pseudo-autosomal regions on the reference genome. These mask regions are listed in Additional files 6, 7, and 8. We applied the same filtering to the NA12878 SV lists obtained from sequencing-based methods. For the complex SVs, we filtered out those located within the pseudo-autosomal regions or overlapping with regions with a ultra-high density of nicking sites, defined as regions spanning 200 kbp or more with at least 333 nicking sites per megabase pair. This density threshold was chosen because it corresponds to an average distance between adjacent nicking sites of 3 kbp, for which it is hard to detect complex SVs accurately.

### Generation of simulated data

We generated simulated data with either only homozygous variants or both homozygous and heterozygous variants. Two steps were involved in both cases, namely a first step for generating genomic sequences with genetic variations introduced into the human reference genome, and a second step for simulating optical maps based on the resulting genomic sequences using the error model described above.

#### Simulated data with only homozygous variants

For the data set with homozygous variants only, we first downloaded the human reference sequence hg38 from the UCSC Genome Browser [41]. We then generated mutations (single nucleotide variants, small and large indels, and complex SVs) on it using pIRS (Profile-Based Illumina Pair-end Reads Simulator) [42]. This software was originally developed for generating short sequencing reads. We took its intermediate file containing the mutated sequence without generating the short reads. In the second step, we used the mutated sequence as input to generate simulated optical maps based on our generative model. The parameter values used in the two steps are shown in Additional file 1: Tables S15 and S16, respectively. The parameter values for the first step were determined based on corresponding estimates from human genomes reported in previous studies [43–45]. The parameter values for the second step were estimated from our actual optical maps by aligning all molecules to the reference sequence using RefAligner, and estimating the parameter values by likelihood maximization. None of these parameter values were made known to our SV detection methods.

#### Simulated data with both homozygous and heterozygous variants

For the data set with both homozygous and heterozygous variants, we generated a diploid genome as follows. It was initialized with our generated haploid genome and the reference genome as the two haplotypes. Then, each variant on the first haploid genome received a probability of *p*_hom_ to be copied to the second haploid genome, resulting in a homozygous variant. Each of the remaining variants, which remained heterozygous, received a probability of *p*_het_ of moving from the first haploid genome to the second. We used *p*_hom_=0.5 and *p*_het_=0.5 in our simulations based on a previous study [46]. As a result of this procedure, the total number of SV loci in this diploid genome was the same as that in the haploid genome.

We then considered the two haploid genomes together as a diploid genome, and used the corresponding DNA sequences as templates to produce OM data using our generative model. The parameter values used in the two steps of the simulation are again shown in Additional file 1: Tables S15 and S16, and 26 additional data sets were generated by changing the false positive rate, false negative rate, and coverage depth, as shown in Additional file 1: Table S2.

### Evaluation metrics of SV calling on simulated data

For the simulated data, we used the known locations of the generated SVs to compute the precision (the fraction of identified SVs that are real) and recall (the fraction of real SVs that are identified) rates of an SV-calling method. An SV call was considered correct if it overlapped the location of a generated SV of the same type.

### Comparison with BioNano Solve

We compared OMSV with the SV caller included in BioNano Solve v3.0 (downloaded from https://bionanogenomics.com/support/software-downloads/), which was the only SV caller for nanochannel-based optical maps with publicly available software. The exact command-line arguments used can be found on the supplementary website.

### Evaluating the performance of OMSV in the ideal situation with no alignment errors

To estimate the performance of OMSV in the ideal situation with no alignment errors, instead of supplying optical map alignments as inputs to OMSV, we provided observed-to-reference distance ratios between neighboring nicking sites directly. For each locus, the number of distance ratios was drawn from a Gaussian distribution estimated based on the coverage depth of the data set. These distance ratios were produced by adding scaling errors to the actual distance ratio of the corresponding allele based on the sizing error parameter of the default simulated data set. The ratio of loci with and without SVs also followed the ratio in the default data set.

### Evaluation metrics of the alignment pipeline with simulated data

We also defined metrics for evaluating the performance of our alignment pipeline. First, an optical map was considered correctly aligned if it was aligned to the correct haplotype of the simulated genome with the aligned location overlapping the actual location from which the optical map was generated. The alignment precision was then defined as the fraction of aligned optical maps that were correctly aligned, and recall was defined as the fraction of generated optical maps that were correctly aligned.

### Integrating and de-duplicating indels from the trio

In the comparisons with the results of the manual checks and the SVs reported in the two previous studies [2, 30], we first integrated the indels from the three individuals. For indels that overlapped, we de-duplicated them by merging them into a larger indel that spanned over all these original indels. For each resulting indel, we considered that it was present for an individual if the individual originally had an indel that overlapped it.

### Definition of Mendelian concordance

For the family trio, a locus was defined as concordant with Mendelian inheritance if the daughter’s genotype could be produced by the genotypes of the father and the mother. When zygosity was not considered, an SV identified from an individual could mean that the individual had the SV in homozygous or heterozygous form. As a result, a Mendelian error was reported only when the daughter had an SV at a locus of a type that both parents did not have. When zygosity was considered, a Mendelian error was reported when the two alleles of the daughter could not have come from the two parents. For this part of the analysis, we considered only loci at which each of the individuals had an SV confidently called or it was highly unlikely that an SV could be called. The former was defined as SVs with at least ten supporting optical maps and a likelihood ratio of at most 10^−6^ for each other hypothesis. The latter was defined as cases in which an SV could not be called even at the loose thresholds of four supporting optical maps and a likelihood ratio of 1.

### Computation of expected Mendelian concordance

To check whether the observed Mendelian concordances of the SVs identified from the trio were consistent with the precision and recall estimates of our simulation, we computed the expected Mendelian concordance as follows. First, we estimated the probabilities *P*(*G*_2_|*G*_1_) where *G*_1_ and *G*_2_ are, respectively, the actual genotype and the genotype called by OMSV, each with possible alleles *A* (the reference allele) and *a* (the alternative allele). The probabilities *P*(*a* *a*|*a* *a*), *P*(*A* *a*|*a* *a*), *P*(*A* *A*|*a* *a*), *P*(*a* *a*|*A* *a*), *P*(*A* *a*|*A* *a*), and *P*(*A* *A*|*A* *a*) were all estimated based on the fraction of homozygous and heterozygous variants generated in our simulated data that were called by OMSV to have the corresponding genotypes. For the remaining three conditional probabilities, *P*(*A* *a*|*A* *A*)=*P*(*A* *A*|*A* *a*)*P*(*A* *a*)/*P*(*A* *A*)≈*P*(*A* *A*|*A* *a*)*P*(*A* *a*), where *P*(*A* *A*|*A* *a*) was again estimated from our simulation result and *P*(*A* *a*) was estimated as half the prior SV probability of the human genome, 8×10^−3^/2 (based on the median total SV size of 20 Mbp per individual reported in Sudmant et al. [2]), assuming an equal probability for homozygous and heterozygous SVs. *P*(*a* *a*|*A* *A*) was estimated in exactly the same way. Finally, *P*(*A* *A*|*A* *A*)=1−*P*(*A* *a*|*A* *A*)−*P*(*a* *a*|*A* *A*).

With all these nine probabilities computed, we charted the probability for each combination of actual and called genotypes of the trio. Specifically, the father, mother, and daughter genotypes were denoted as a triple. For example, (*AA,aa,Aa*) represents where the father has the reference genotype, the mother has an SV in homozygous form, and the daughter has the SV in heterozygous form. The probability for an actual genotype combination *C*_1_ to be called as a genotype combination *C*_2_ was calculated as the product of the three corresponding conditional probabilities, assuming the SV-calling errors of the three individuals are independent. For example, *P*((*AA,AA,aa*)|(*AA,aa,Aa*))=*P*(*AA*|*AA*)*P*(*AA*|*aa*)*P*(*aa*|*Aa*).

When zygosity was considered, the actual genotype combination must come from the set of 15 combinations concordant with Mendelian inheritance, *O*={(*AA,AA,AA*), (*AA,Aa,AA*), (*AA,Aa,Aa*), (*AA,aa,Aa*), (*Aa,AA,AA*), (*Aa,AA,Aa*), (*Aa,Aa,AA*), (*Aa,Aa,Aa*), (*Aa,Aa,aa*), (*Aa,aa,Aa*), (*Aa,aa,aa*), (*aa,AA,Aa*), (*aa,Aa,Aa*), (*aa,Aa,aa*), (*aa,aa,aa*)}. The overall expected Mendelian concordance rate was then calculated as ${\sum \nolimits }_{C_{1}\in O} \left [ {P}(C_{1}){\sum \nolimits }_{C_{2}\in O} {P}(C_{2}|C_{1})\right ]$. We estimated the prior probabilities *P*(*C*_1_) by the number of times such genotype combination was called by OMSV in the trio data.

When zygosity was ignored, the expected Mendelian concordance rate was calculated as

$$ 1 - {\sum\nolimits}_{C_{1}\in S} {P}(C_{1})\! \left[{P}(AA,AA,Aa|C_{1})+{P}(AA,AA,aa|C_{1})\right]\!. $$

### Comparing with sequencing-based results for NA12878 SVs

We lifted over the SV lists of NA12878 from Parikh et al. [30] and Sudmant et al. [2] from hg19 to hg38. We then filtered both these lists and our list of SVs by removing SVs with a size smaller than 2000 bp or overlapping the mask regions. The remaining SVs on the three lists were then compared.

### Production of optical maps from C666-1

#### High-molecular-weight DNA extraction

The C666-1 cell line was washed with phosphate-buffered saline (PBS) and spun down to a pellet. Next, 10^6^ cells/mL were obtained upon resuspension in PBS, and embedded in 1.5 % low-melting agarose plugs in 0.5 × TBE (Tris-Borate-EDTA) (CHEF Genomic DNA Plug Kit, Bio-Rad). Subsequent handling of the DNA followed BioNano Genomics recommended protocols. The agarose plugs were incubated with proteinase K with lysis buffer at 50 °C overnight. The plugs were washed with a wash buffer to stabilize the DNA in the plugs, and the quality was assessed using pulsed-field gel electrophoresis. A plug was then washed with TE (Tris-EDTA) buffer and melted at 70 °C. After being solubilized with 0.4 U of GELase (Epicentre), the purified DNA was subjected to 2.5 h of drop-dialysis and was shredded by nine strokes of gentle pipetting. The viscous DNA was allowed to equilibrate overnight at room temperature to increase the homogeneity. It was then quantified using a Qubit Broad Range dsDNA Assay Kit (Life Technologies).

#### DNA labeling

The DNA was labeled using the IrysPrep Reagent Kit (BioNano Genomics). Specifically, 300 ng of purified genomic DNA was nicked with 0.3 U of nicking endonuclease Nt.BspQI (New England BioLabs, NEB) at 37 °C for 2 h in buffer BNG3. The nicked DNA was labeled with a fluorescent-dUTP nucleotide analog using Taq polymerase (NEB) for 1 h at 72 °C. After labeling, the nicks were ligated with Taq ligase (NEB) in the presence of dNTPs. The backbone of fluorescently labeled DNA was counterstained with YOYO-1 (BioNano Genomics IrysPrep Reagent Kit).

#### Data collection and assembly

The DNA was loaded onto a BioNano Genomics IrysChip and linearized and visualized by the Irys system. The DNA backbone length and locations of fluorescent labels along each molecule were detected using the Irys software. Single-molecule maps were assembled de novo into genome maps using the IrysSolve software tools developed at BioNano Genomics [15].

### Comparing C666-1 indels with human segmental duplications

We downloaded segmental duplication regions in the human reference genome hg38 from the UCSC Genome Browser, and annotated them with gene information for those overlapping gene exons. We then compared the C666-1 indels identified by OMSV with these segmental duplication regions to look for overlaps.

### Identifying SVs from C666-1 using short reads

We used the default settings of Manta and Pindel to identify SVs from the sequencing data of C666-1. We considered only large (>2 kbp) SVs supported by at least 20 reads/read pair.

### Selection of C666-1 SVs for experimental validation

We selected SVs identified by OMSV from C666-1 cells for experimental validation based on the following two criteria: (1) We selected only insertions and complex SVs, since these SVs are particularly difficult to identify and their sizes are difficult to determine from sequencing reads alone. (2) We selected SVs with primers that could be designed from non-repeat regions and which would lead to amplicons analyzable by PCR. The selected SVs and the designed primers are listed in Additional file 1: Tables S10–S13.

### Integrating sequencing reads to infer precise break points and inserted sequences

For each homozygous insertion identified by OMSV from C666-1 that occurs within the region [*o*_1_,*o*_2_] of the human reference genome sequence hg38 with an estimated size of *s*, we performed the following steps (Additional file 1: Figure S9, Table S10):

1. Construct a tentative C666-1 sequence by replacing the region [*o*_1_,*o*_2_] by *x* copies of *N* (i.e., unknown) nucleotides, where *x*=*o*_2_−*o*_1_+*s* for an insertion and *x*=*o*_2_−*o*_1_−*s* for a deletion.
2. Use GapCloser [47] to infer the actual sequence of this *N* region based on a local assembly of sequencing reads and the flanking sequences, which may or may not resolve all the *N*’s.
3. Align sequencing reads to the region [*o*_1_,*o*_2_] of the reference sequence using BWA [48], visualizing only read pairs with both sides aligned using IGV [49].
4. Align sequencing reads to the inferred C666-1 sequence using BWA, visualizing only read pairs with both sides aligned.
5. Use the alignment results to evaluate the confidence of the SV, the break points, and the inserted sequences for insertions.

## Additional files

Additional file 1 Supplementary tables and figures. Containing Tables S1–S16 and Figures S1–S11. (PDF 1930 kb)

Additional file 2 SV lists from the CEU (Northern Europeans from Utah) trio. This file provides the SVs identified by OMSV from the CEU trio. The first three sheets list the indels identified from NA12878, NA12891, and NA12892, respectively. The fourth sheet lists the union of these three lists. The fifth sheet lists all the sites with multiple indels called at the same site (two insertions, two deletions, or one insertion and one deletion). The sixth sheet lists the high-confidence indels and non-indels for evaluating Mendelian concordance. The last sheet lists the complex SVs. (XLSX 570 kb)

Additional file 3 SV list from the C666-1 cell line. This file provides the SVs identified by OMSV from the C666-1 cell line. The first sheet lists the indels identified. The second sheet lists all the sites with multiple indels called at the same site (two insertions, two deletions, or one insertion and one deletion). The third sheet lists the complex SVs. (XLSX 110 kb)

Additional file 4 Overlapping of C666-1 indels with segmental duplications. This file provides the overlap of C666-1 indels identified by OMSV with human segmental duplications. The first three columns show the genomic location of the SVs. The fourth column shows the SV type. The fifth and sixth columns show the overlapping segmental duplications (if any) and the genes of which the exons overlap the segmental duplications (if any). (XLSX 72 kb)

Additional file 5 Case studies of complex SVs of C666-1. This file provides visualizations of selected cases of complex SVs identified by OMSV from C666-1. (PDF 487 kb)

Additional file 6 Fragile sites in the in silico map based on hg38. This file provides the locations of fragile sites in the human reference genome hg38. (BED 90 kb)

Additional file 7 Gaps in hg38. This file provides the locations of unspecified nucleotides (N’s) in the human reference genome hg38. (BED 18 kb)

Additional file 8 Pseudo-autosomal regions in hg38. This file provides the locations of pseudo-autosomal regions in the human reference genome hg38. (BED 0.078 kb)

## Abbreviations

bp: Base pair
CNV: Copy number variation
EBV: Epstein–
Barr virus; IF: Intrinsically feasible
Indel: Insertion or deletion
kbp: Kilobase pair
Mbp: Megabase pair
OM: Optical mapping
PBS: Phosphate-buffered saline
PCR: Polymerase chain reaction
SV: Structural variation
