# Supplementary material for: OMSV enables accurate and comprehensive identification of large structural variations from nanochannel-based single-molecule optical maps
Source: Genome Biol. 2017 Dec 1;18:230. doi: 10.1186/s13059-017-1356-2 (PMC5709945; doi:10.1186/s13059-017-1356-2)
Supplement: Supplementary file 1 — Supplementary tables and figures. Containing Tables S1–S16 and Figures S1–S11. (PDF 1930 kb) [file 13059_2017_1356_MOESM1_ESM.pdf]

Supplementary tables

Publication	SV types supported	Zygosity call?	Assembly-based?	Software availability
Teague et al. (2010) [18]	Indels	No	Yes	Not publicly available
Ray et al. (2013) [17]	Indels	No	Yes	Not publicly available
Cao et al. (2014) [15]	Indels/inversions	No	Yes	Predecessor of BioNano Solve
Gupta et al. (2015) [51]	Indels	No	Yes	Not publicly available
Mak et al. (2016) [28]	Indels/inversions	No	Yes, also a module based on alignment	Predecessors of BioNano Solve and OMSV

Table S1 Existing SV calling methods based on optical mapping.

Genome	Optical maps generated	Genome coverage	FP	FN	Avg. optical map length (bp)	Avg. nicking sites per map	Density of nicking sites (per Mbp)	Optical maps aligned	Alignment rate
Haploid	1,500,000	100	1.2E-5	1.2E-1	200,780	22.9	114.0	1,203,728	80.2%
Diploid	1,500,000	100	1.2E-5	1.2E-1	200,814	22.9	114.0	1,214,539	81.0%
Diploid	300,000	20	1.2E-5	1.2E-1	200,622	22.9	114.1	243,006	81.0%
Diploid	500,000	33	1.2E-5	1.2E-1	200,576	22.9	114.1	404,805	81.0%
Diploid	700,000	47	1.2E-5	1.2E-1	200,681	22.9	114.1	566,912	81.0%
Diploid	900,000	60	1.2E-5	1.2E-1	200,679	22.9	114.1	728,672	81.0%
Diploid	1,100,000	73	1.2E-5	1.2E-1	200,771	22.9	114.0	890,816	81.0%
Diploid	1,300,000	87	1.2E-5	1.2E-1	200,796	22.9	114.0	1,052,735	81.0%
Diploid	1,700,000	113	1.2E-5	1.2E-1	200,820	22.9	114.0	1,376,494	81.0%
Diploid	1,900,000	127	1.2E-5	1.2E-1	200,793	22.9	114.0	1,538,631	81.0%
Diploid	2,100,000	140	1.2E-5	1.2E-1	200,812	22.9	114.0	1,700,717	81.0%
Diploid	2,300,000	153	1.2E-5	1.2E-1	200,820	22.9	114.0	1,862,534	81.0%
Diploid	2,500,000	167	1.2E-5	1.2E-1	200,786	22.9	114.0	2,024,415	81.0%
Diploid	1,500,000	100	0	1.2E-1	200,713	20.9	104.1	1,282,369	85.5%
Diploid	1,500,000	100	1.2E-8	1.2E-1	200,791	20.9	104.1	1,282,268	85.5%
Diploid	1,500,000	100	1.2E-7	1.2E-1	200,806	20.9	104.1	1,281,988	85.5%
Diploid	1,500,000	100	1.2E-6	1.2E-1	200,785	21.1	105.1	1,276,595	85.1%
Diploid	1,500,000	100	3.0E-5	1.2E-1	200,772	25.9	129.0	1,096,354	73.1%
Diploid	1,500,000	100	6.0E-5	1.2E-1	200,801	30.7	152.9	1,018,500	67.9%
Diploid	1,500,000	100	9.0E-5	1.2E-1	200,834	35.3	175.8	1,303,880	86.9%
Diploid	1,500,000	100	1.2E-4	1.2E-1	200,816	39.6	197.2	1,366,980	91.1%
Diploid	1,500,000	100	1.2E-5	0	200,538	25.3	126.2	1,309,947	87.3%
Diploid	1,500,000	100	1.2E-5	1.2E-4	200,525	25.3	126.2	1,310,001	87.3%
Diploid	1,500,000	100	1.2E-5	1.2E-3	200,564	25.3	126.2	1,309,055	87.3%
Diploid	1,500,000	100	1.2E-5	1.2E-2	200,564	25.1	125.1	1,303,178	86.9%
Diploid	1,500,000	100	1.2E-5	2.4E-1	201,321	20.5	101.8	1,037,081	69.1%
Diploid	1,500,000	100	1.2E-5	3.6E-1	202,626	18.0	88.8	768,478	51.2%
Diploid	1,500,000	100	1.2E-5	4.8E-1	205,510	15.7	76.4	444,795	29.7%

Table S2 Statistics of the simulated optical maps. FP and FN refer to the rates for a fake nicking site to be observed and a real nicking site to be unobserved, respectively. The first two rows show the statistics of the haploid and diploid data sets based on the default setting, and the other rows show the settings with different genome coverage, FP and FN values.

Genome Genome	Homozygous insertions	Homozygous deletions	Heterozygous insertions	Heterozygous deletions	Complex	Total
Haploid	936	911	0	0	983	2830
Diploid	485	467	451	444	983	2830

Table S3 Statistics of SVs in the simulated data sets. All 27 diploid data sets listed in Table S2 were generated based on the same diploid genome with the SV profile shown here. The number of complex SVs generated is larger than that of a typical human sample, to test OMSV's ability to identify complex SVs.

Step	Time needed (hours)	
	Haploid genome	Diploid genome
OMBlast alignment (using 1 thread)	225	225
OMBlast alignment (using 64 threads)	3.47	3.52
RefAligner alignment (using 1 thread)	49	50
RefAligner alignment (using 64 threads)	0.77	0.79
SV calling (using 1 thread)	1.24	1.24
Total (using 1 thread)	226	226
Total (using 64 threads)	4.71	4.76

Table S4 Running time of OMSV on simulated data with 100x coverage of the human genome. The total amount of time is defined as the maximum time for the two alignment methods plus the time for SV calling.

Samples	Optical maps generated	Avg. optical map length (bp)	Avg. nicking sites per map	Density of nicking sites (per Mbp)	Optical maps aligned	Alignment rate
NA12878	1,540,247	207,926	22.5	108.2	1,264,390	82.1%
NA12891	1,481,578	214,366	24.4	113.8	1,205,487	81.4%
NA12892	2,065,938	184,264	19.8	107.5	1,641,813	79.5%

Table S5 Statistics of the optical maps produced from the family trio.

Sample	SV type	On autosomes	On sex chromosomes*	X error	Y error
NA12878 (daughter)	Insertion	538	27	N/A	0
	Deletion	523	25	N/A	0
	Multiple	8	1	N/A	0
	CNV	29	1	N/A	0
	Medium Inversion	30	0	N/A	0
	Large Inversion	22	5	N/A	0
	Intra-chromosomal Translocation	1	0	N/A	0
	Inter-chromosomal Translocation	1	0	N/A	0
	Total	1,152	59	N/A	0
NA12891 (father)	Insertion	573	31	7	2
	Deletion	500	22	8	1
	Multiple	7	0	N/A	N/A
	CNV	22	6	N/A	N/A
	Medium Inversion	27	1	N/A	N/A
	Large Inversion	25	4	N/A	N/A
	Intra-chromosomal Translocation	0	0	N/A	N/A
	Inter-chromosomal Translocation	1	0	N/A	N/A
	Total	1,155	64	15	3
NA12892 (mother)	Insertion	536	20	N/A	0
	Deletion	477	21	N/A	0
	Multiple	6	0	N/A	N/A
	CNV	45	3	N/A	N/A
	Medium Inversion	21	0	N/A	N/A
	Large Inversion	31	6	N/A	N/A
	Intra-chromosomal Translocation	2	0	N/A	N/A
	Inter-chromosomal Translocation	45	5	N/A	N/A
	Total	1,163	55	N/A	3

Table S6 Statistics of SVs called from the optical maps produced from each member of the trio. The "Multiple" SV type corresponds to a locus with multiple indels called at the same site (two insertions, two deletions, or one insertion and one deletion). These cases are not included in the counts of the "Insertion" and "Deletion" cases. X error includes SVs called in the non-pseudo-autosomal regions of the X chromosome as heterozygous from a male sample. Y error includes SVs called in the non-pseudo-autosomal regions of the Y chromosome either from a female sample or in heterozygous form from a male sample. Since OMSV does not determine the zygosity of complex SVs, they were not included in the calculation of X and Y errors that involved zygosity. An inter-chromosomal translocation is counted as appearing on a sex chromosome if either of the two chromosomes involved is a sex chromosome. *Pseudo-autosomal regions are excluded.

Sample	Total number of SVs called	Intersection with with manual checking list	Validated by manual checking		Validation rate	
			Ignoring zygosity	Considering zygosity	Ignoring zygosity	Considering zygosity
NA12878	991	726	705	527	0.97	0.73
NA12891	1007	696	669	516	0.96	0.74
NA12892	926	642	615	471	0.96	0.73

Table S7 Accuracy of the SVs called by OMSV based on the manual checking results in Mak et al. The SVs from the three individuals were integrated and de-duplicated, and then the SVs contained in each individual were extracted from the resulting list, before comparing with the manual checking results.

Optical maps generated	Avg. optical map length (bp)	Avg. nicking sites per map	Density of nicking sites (per Mbp)	Optical maps aligned	Alignment rate
1,644,102	244,075	22.8	93.4	1,129,075	68.7%

Table S8 Statistics of the optical maps produced from the C666-1 cell line.

Sample	SV type	On autosomes	On sex chromosomes*	X error	Y error
C666-1 (male)	Insertion	527	16	6	0
	Deletion	262	5	0	0
	Multiple	3	0	N/A	N/A
	CNV	66	2	N/A	N/A
	Medium inversion	24	4	N/A	N/A
	Large inversion	10	3	N/A	N/A
	Intra-chromosomal translocation	2	0	N/A	N/A
	Inter-chromosomal translocation	4	0	N/A	N/A
	Total	898	30	6	0

Table S9 Statistics of SVs called from the C666-1 cell line optical maps. The "Multiple" SV type corresponds to a locus with multiple indels called at the same site (two insertions, two deletions, or one insertion and one deletion). These cases are not included in the counts of the "Insertion" and "Deletion" cases. X error and Y error respectively includes SVs called in the non-pseudo-autosomal regions of the X and Y chromosome as heterozygous. Since OMSV does not determine the zygosity of complex SVs, they were not included in the calculation of X and Y errors. An inter-chromosomal translocation is counted as appearing on a sex chromosome if either of the two chromosomes involved is a sex chromosome. *Pseudo-autosomal regions are excluded.

SV ID	I_{o1}	I_{o2}	I_{o3}	I_{o4}	I_{o5}	I_{o6}	I_{o7}
Chr	1	3	8	12	14	15	16
o_1	10,969,439	154,172,724	21,505,975	40,144,059	104,496,071	74,214,211	86,985,730
o_2	10,971,543	154,184,477	21,525,476	40,149,705	104,499,324	74,220,313	86,988,880
s	2,501	3,273	2,050	2,405	7,222	2,217	2,012
Primer location							
Left primer	10,969,341-10,969,363	154,179,761-154,179,783	21,516,348-21,516,372	40,145,694-40,145,715	104,498,683-104,498,705	74,215,116-74,215,138	86,986,707-86,986,727
Right primer	10,971,633-10,971,656	154,181,240-154,181,263	21,517,547-21,517,570	40,146,848-40,146,870	104,499,061-104,499,084	74,218,504-74,218,526	86,987,474-86,987,496
Predicted PCR product size							
With SV	4,817	4,776	3,273	3,562	7,624	5,628	2,780
Without SV	2,316	1,503	1,223	1,157	402	3,411	768
Detected by sequencing-based SV caller?							
Manta	Yes	Yes	Yes	No	No	Yes	No
Pindel	No	No	No	No	No	No	No
b_1	10,971,095-	154,180,619-	21,516,373-	40,145,895-	104,498,838-	74,216,616-	86,986,874-
b_2	10,971,077	154,180,621	21,517,291	40,146,766	104,498,911	74,216,611	86,986,968

Table S10 List of homozygous insertions identified by OMSV from C666-1 that underwent experimental validations. Definitions of o_1, o_2, s, b_1 and b_2 are given in Figure S9. PCR product sizes were predicted by considering both primer locations and insertion size s determined by OMSV.

SV ID	I_{e1}	I_{e2}	I_{e3}	I_{e4}	I_{e5}	I_{e6}	I_{e7}
Chr	2	4	5	5	20	1	4
o_1	22,961,852	37,948,238	9,967,483	137,676,345	61,555,087	223,473,487	96,500,882
o_2	22,969,321	37,960,828	9,972,346	137,689,062	61,561,856	223,487,948	96,507,004
s	6,190	4,085	3,496	4,904	5,069	2,428	3,620
Primer location							
Left primer	22,961,879-22,961,901	37,949,749-37,949,772	9,970,229-9,970,251	137,678,394-137,678,415	61,560,697-61,560,717	223,474,377-223,474,399	96,501,977-96,502,001
Right primer	22,963,770-22,963,792	37,955,267-37,955,289	9,971,538-9,971,560	137,679,399-137,679,421	61,561,964-61,561,985	223,479,822-223,479,844	96,502,754-96,502,777
Predicted PCR product size							
With SV	8,104	9,626	4,828	7,232*	6,358	7,896	4,421
Without SV	1,914	5,541	1,332	1,028	1,289	5,468	801
Detected by sequencing-based SV caller?							
Manta	No	Yes (het.)	Yes (het.)	Yes (het.)	No	No	Yes (het.)
Pindel	No	No	No	No	No	No	No
b_1	22,962,381-	37,950,488-	9,971,225-	137,682,429-	61,559,538-	Unable to infer	Unable to infer
b_2	22,962,381	37,950,488	9,971,225	137,682,429	61,559,499	Unable to infer	Unable to infer

Table S11 List of heterozygous insertions identified by OMSV from C666-1 that underwent experimental validations. Definitions of o_1, o_2, s, b_1 and b_2 are given in Figure S9. PCR product sizes were predicted by considering both primer locations and insertion size s determined by OMSV. In the Manta predictions, het. denotes that the SV was predicted to be heterozygous. *The predicted PCR product size of I_{e4} is not equal to the summation of the size without SV and s, because GapCloser's result reveals an extra deletion of around 1,300bp between o_1 and o_2 but outside the designed primer pair. As a result, the expected PCR product size without the SV is not affected (since the deletion is outside the primer pair) and is equal to the span of the genomic region covered by the two primers, but the estimated insertion size s should be increased by 1,300bp since it was originally inferred by OMSV without knowing that the two defining nicking sites were actually 1,300bp closer to each other in C666-1 as compared to the reference genome.

SV ID	C_1	C_2	C_3
Type	Inter-trans.	Intra-trans.	Inversion
Chr 1st	5	8	X
o_1	77,697,732	22,574,642	149,634,449
Translocated segment 1st	left of o_1	left of o_1	
Chr 2nd	8	8	X
o_2	27,323,114	30,293,029	149,727,161
Translocated segment 2nd	left of o_2	right of o_2	
Primer location			
Primer pair 1			
Left primer	chr5:77,933,545-chr5:77,933,567	22,570,830-22,570,852	149,652,660-149,652,682
Right primer	chr8:27,559,009-chr8:27,559,031	30,262,222-30,262,244	149,748,923-149,748,945
Primer pair 2 (for inversions)			
Left primer			149,654,409-149,654,431
Right primer			149,750,609-149,750,631
Predicted PCR product size			
Primer pair 1			
With SV	800-1,700	400-1,300	1,500-2,800
Without SV	No product	No product	No product
Primer pair 2 (for inversions)			
With SV			1,500-2,800
Without SV			No product
Detected by sequencing-based SV caller?			
Manta	Yes	Yes (Del.)	No
Pindel	No	No	No
b_{11}	chr5:77,934,208-	22,571,087-	149,653,932-
b_{12}	chr5:77,934,308	22,571,287	149,654,132
b_{21}	chr8:27,559,341-	30,261,884-	149,748,939-
b_{22}	chr8:27,559,441	30,261,984	149,749,439

Table S12 List of complex SVs identified by OMSV from C666-1 that underwent experimental validations. Inter-trans. and Intra-trans. refer to inter-chromosomal translocation and intra-chromosomal translocation, respectively. Chr 1st and Chr 2nd are the chromosomes of the first and second break points, which are different for inter-chromosomal translocations. Definitions of o_1 and o_2 and locations of the break points estimated by OMSV. [b_{11}, b_{12}] and [b_{21}, b_{22}] are the approximate break point locations estimated by sequencing reads. PCR product sizes were predicted by considering both the distance between the defining nicking sites and the locations of the designed primers. In the Manta predictions, del. denotes that the SV was predicted to be a deletion.

SV ID	Primer	Primer sequence
I_{o1}	Left	GACATCCAATGCTTTCCTACTCC
	Right	TCAAGTCAGGAAGGAAAGAGACAC
I_{o2}	Left	TGGATGTTGGTTACTGGGAATGG
	Right	CCAGATAAGTGGCAGCGAAGTATG
I_{o3}	Left	CAGGCAGTTCTGGATGCATTGTCAC
	Right	GTGACTTGCCTGATCAACAGAATG
I_{o4}	Left	CAAGGTGAAACCCCGTCTCTAC
	Right	GTTGTCCTCTTGTTTGAACCTGC
I_{o5}	Left	AAAAGGGATTCTCACACTCTCGG
	Right	AGATCAGTATTCAGGCTCAGTGTG
I_{o6}	Left	TAGCCAGTCTGCAGGATGAGTAG
	Right	CATCATGCTGCCCGTCATTCTTG
I_{o7}	Left	GTAAGTGTGCTATTGGCTGTG
	Right	GGACTGGTTAAATGGTGATTAGG
I_{e1}	Left	TTACCTGAGACACATAGACTGGG
	Right	TGGTAGCCAGACACTGTAATAGG
I_{e2}	Left	ATGTAGCAACTATTCAACTCTGCC
	Right	GGGAATCTCTCATTAAGTCTGCG
I_{e3}	Left	AACAGCCCAAACAACTCTATAGG
	Right	TAGCAGTGTTGTGAGATACCAGC
I_{e4}	Left	GGCCATCATCCTGCTATTAGAG
	Right	CAGAGATGTTGGTGCTGGTTTGC
I_{e5}	Left	TTAGCTTCCAGGAACGTGAGC
	Right	GAAGGTCCCTCTCTCTCTCTGG
I_{e6}	Left	AGAGGGAAAGAAGGAAGGGAAGC
	Right	TGTTAAAGCTGGAGGGAAGTAGG
I_{e7}	Left	CACTCTCTTCAATAAATAGTGCTGG
	Right	AGTCCAGAGCACTAGATAAGAATG
C_1	Left	CCAAATTCATGGGGAGGGAACAC
	Right	CTGAGTGGAACCTGTCAATGCTG
C_2	Left	GAATGAGACAGCCCAGATAAGGC
	Right	CTACCTTCAGCATCAGATCCAGG
C_3 (pair 1)	Left	CTTGCTATCCTCTGACCCCTGAG
	Right	GTAACATAAGGCAGGAGATATGG
C_3 (pair 2)	Left	CCTACGATCACTGGCCAGCATAC
	Right	GGTTGACAGCATGGGCAGAAACG

Table S13 Primer sequences used in the PCR validation experiments of the insertions identified by OMSV from C666-1.

Module	Parameter	Estimation method/default value
Site	False positive rate (fp)	1E-5, estimated by RefAligner
Site	False negative rate (fn)	0.125, estimated by RefAligner
Site	P-value threshold	1E-9
Site, Size	Likelihood ratio threshold	1E-6
Site, Size	Minimum covering optical maps (M_{min})	15 for Site, 10 for Size
Size	Minimum optical maps from each chromosome in heterozygous calls (k_{min})	$\max(5, 0.4M)$, where M is the number of covering optical maps
Size	Global distance ratio location parameter (r_0)	1.0096, by MLE estimate of Matlab fminsearch function
Size	Global distance ratio scale parameter (γ)	0.0291, by MLE estimate of Matlab fminsearch function
Size	Minimum distance change (δ)	$\max(2000, 0.05d_0)$, where d_0 is the distance on the reference

Table S14 Parameter values used in the SV detection modules of OMSV. Abbreviations of SV types: "Site" – extra/missing sites; "Size" – SVs with large size changes; "Complex" – complex SVs.

	SNP (The 1000 Genomes Project Consortium, 2010)	Indel (Lu et al., 2012)	Insertion/Deletion/Inversion (Pang et al., 2010)
Rate	1E-3	1E-4	1E-6
Number generated	$2,910,896$	$298,715$	$2,942$
Size range (bp)	1	$2-70$	$5,000-100,000$

Table S15 Parameter values used in simulating a haploid genome (i.e., step 1 of simulation). The cited publications are the references for the chosen values.

Parameter	Symbol	Value used	Rationale
Number of optical maps	n	1,500,000	To get 100x coverage
Minimum DNA fragment size	l_0	100,000	Typical experimental protocol
Average DNA fragment extra size	μ_l	100,000	Typical size in real data
Restriction enzyme		Nt.BspQI	Reasonable restriction site density
False negative rate	f_-	0.12	RefAligner's estimate from real data
False positive rate	f_+	1.2E-5	RefAligner's estimate from real data
Position parameter of sizing error	o_α	1.00	Maximum likelihood estimate from real data
Scale parameter of sizing error	s_α	0.02	Maximum likelihood estimate from real data
Imaging resolution	$d_{\frac{1}{2}}$	700	Observation from real data
Measurement error	e	50	Pixel resolution of optical map images

Table S16 Parameter values used in generating simulated optical mapping data from a haploid genome (i.e., step 2 of simulation).

Supplementary figures

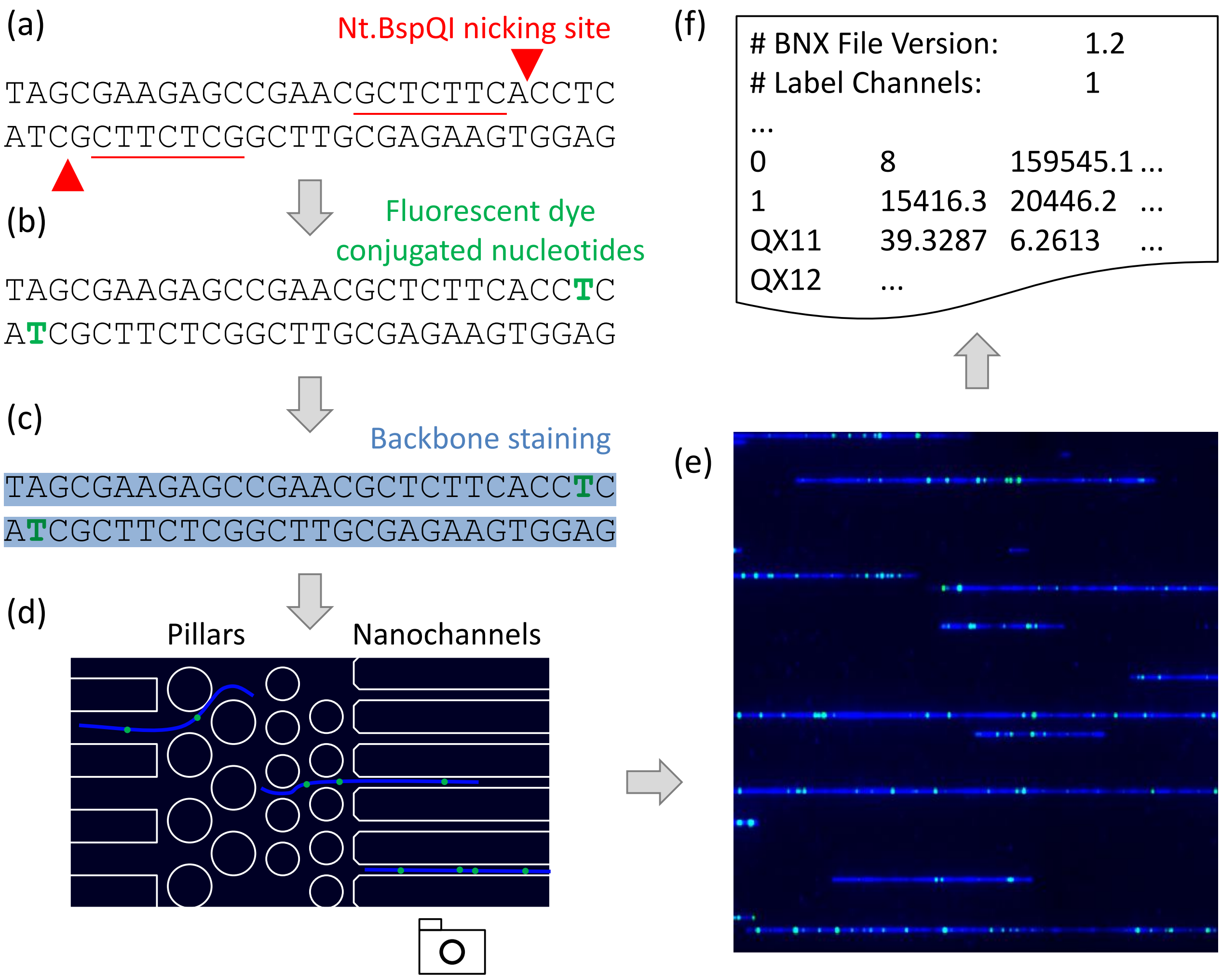

Figure S1 The nanochannel-based single-molecule optical mapping method. (a) DNA molecules are recognized by a nicking enzyme (Nt.BspQI as example here) at the restriction sites (underlined) and nicks are introduced on a single strand (red triangles). (b) The nicks are repaired, with fluorescent dye conjugated nucleotides (green) replacing nearby thymines. (c) The DNA backbone is fluorescently stained (blue). (d) The DNA molecules are loaded onto flow cells, uncoiled in the gradient pillars, and finally linearized in nanochannels, at which they are imaged by high-resolution fluorescent microscopy. (e) The resulting images show the DNA molecules and the labels at the nicking sites. (f) The final output is a file that contains information about the experiment and the molecules, including the locations of the labels on each molecule and quality scores.

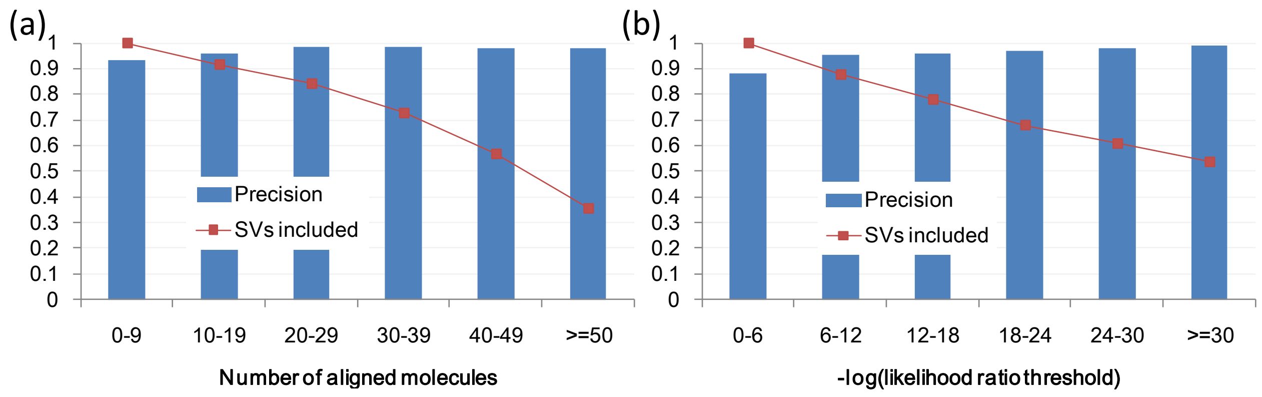

Figure S2 Precision of OMSV and the fraction of SVs covered (a) for various numbers of molecules aligned to the SV regions and (b) with different values of the likelihood ratio threshold of OMSV, based on the diploid data set with default setting.

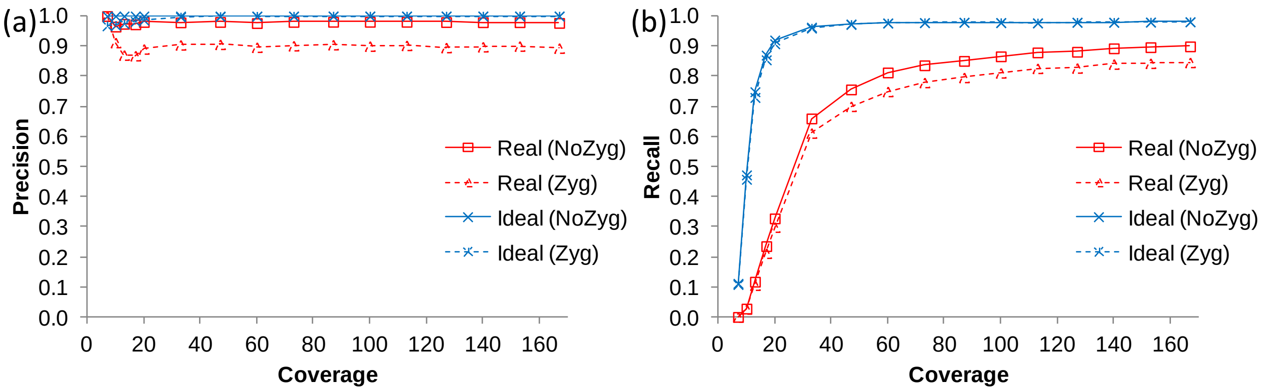

Figure S3 Precision (a) and recall (b) of OMSV with ("Real") or without ("Ideal") actual alignments, based on diploid data sets with various depths of coverage.

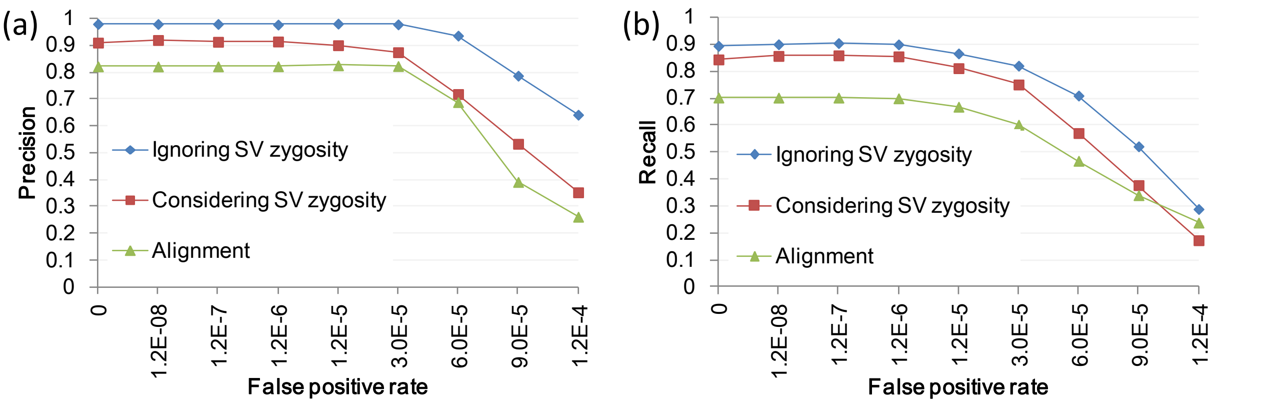

Figure S4 Precision (a) and recall (b) of OMSV and its alignment pipeline, based on diploid data sets with various false positive rates of nicking sites. The Alignment series shows the precision/recall of optical map alignments, which is observed to correlate with SV calling precision/recall.

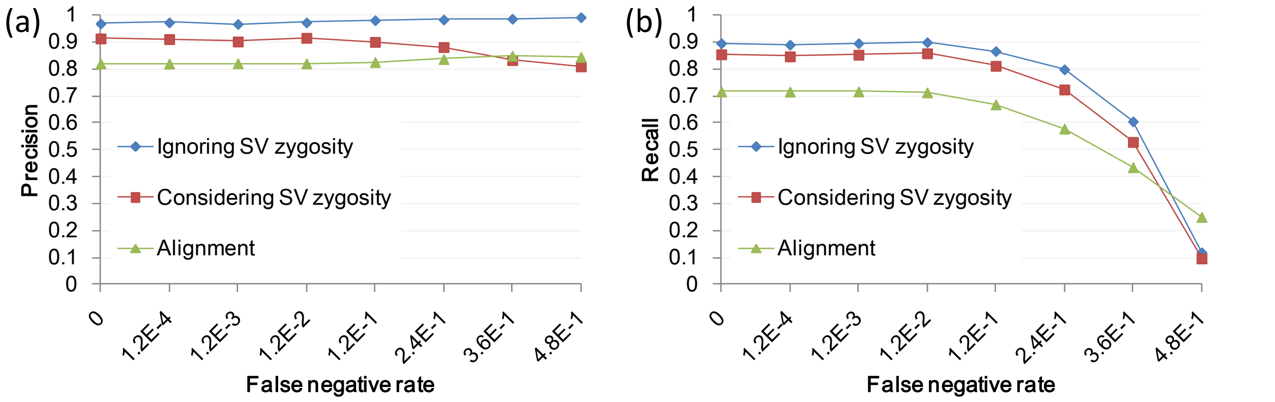

Figure S5 Precision (a) and recall (b) of OMSV and its alignment pipeline, based on diploid data sets with various false negative rates of nicking sites.

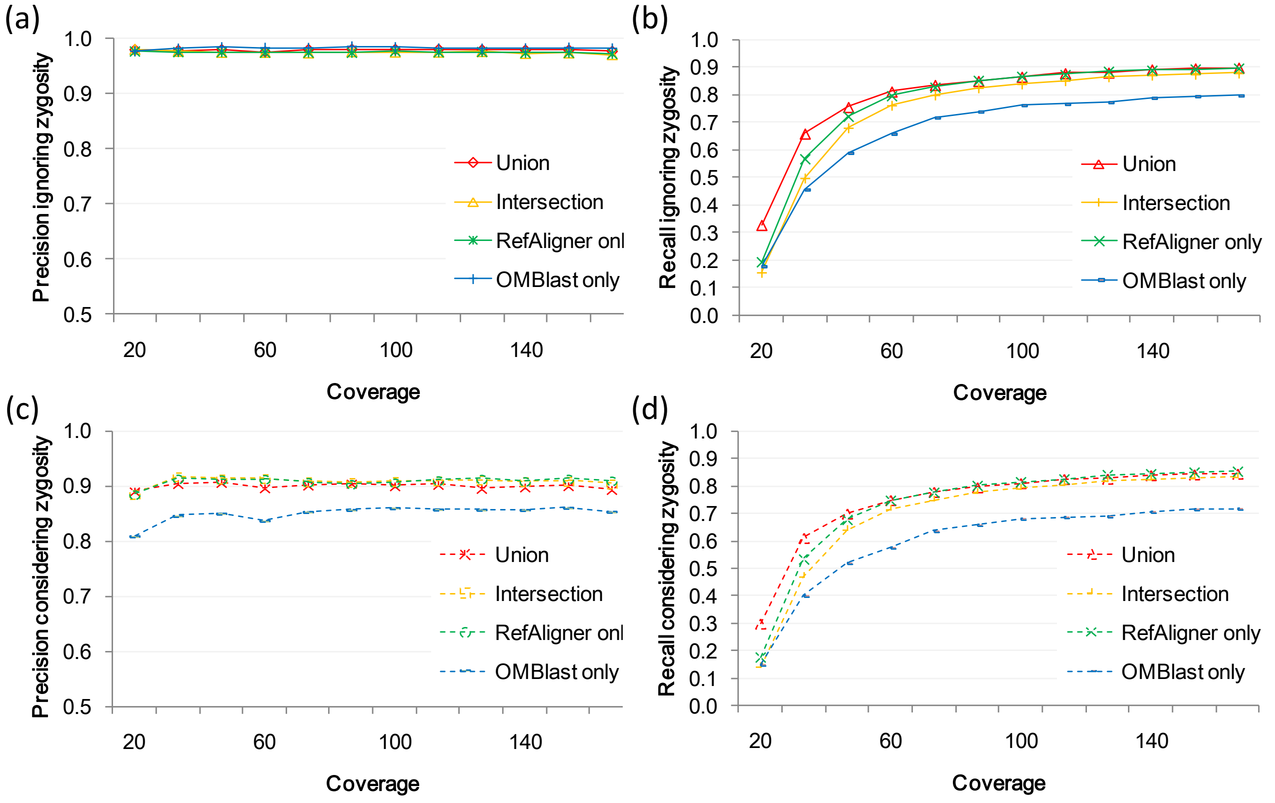

Figure S6 Performance of OMSV with optical map alignments provided by either a single aligner, the intersection of both aligners, or the union of them.

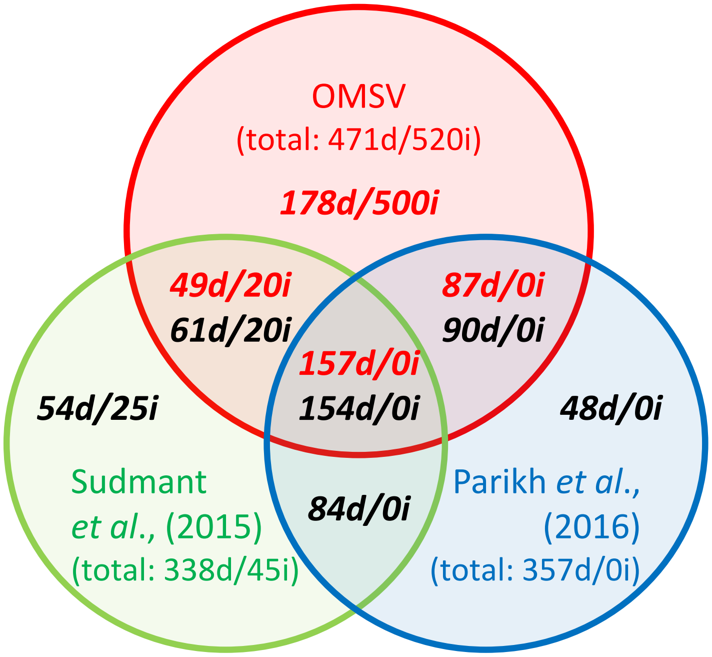

Figure S7 Comparison of indels detected by OMSV from NA12878 with two published lists obtained by sequencing-based methods. The SVs from the three individuals of the trio were integrated and de-duplicated, and then the ones contained in NA12878 were extracted from the resulting list. In each region, the numbers of deletions and insertions are shown as del/ins, where the numbers of indels identified by OMSV are in red while the numbers of indels reported in the previous studies are in black. Since an indel on one list could overlap multiple indels on another list, the red and black numbers in the same region are not necessarily the same. Loci with both an insertion and a deletion identified were not included in this comparison since the two published lists did not contain such cases.

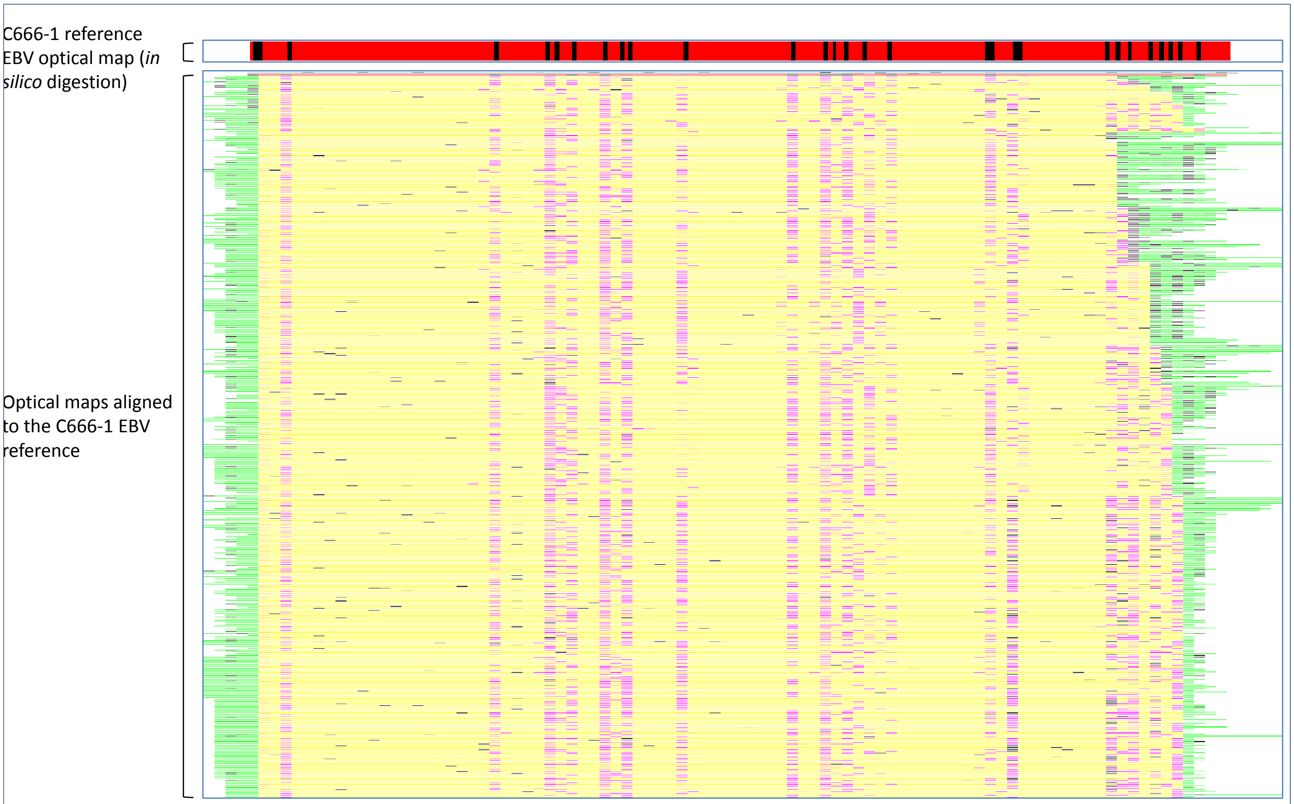

Figure S8 Optical maps produced from C666-1 aligned to the EBV reference. In the reference (top red horizontal bar), the vertical black lines mark the locations of the expected nicking site locations based on the EBV genome sequence in C666-1. In the optical map section (bottom), each horizontal line corresponds to one optical map aligned to the EBV genome, where the aligned parts without nicking site signals are marked in yellow, the aligned nicking sites are marked in pink, extra/missing nicking sites detected are marked in black, and unaligned parts are marked in green. The unaligned parts at the two ends are due to the circular nature of episomal EBV genome in C666-1.

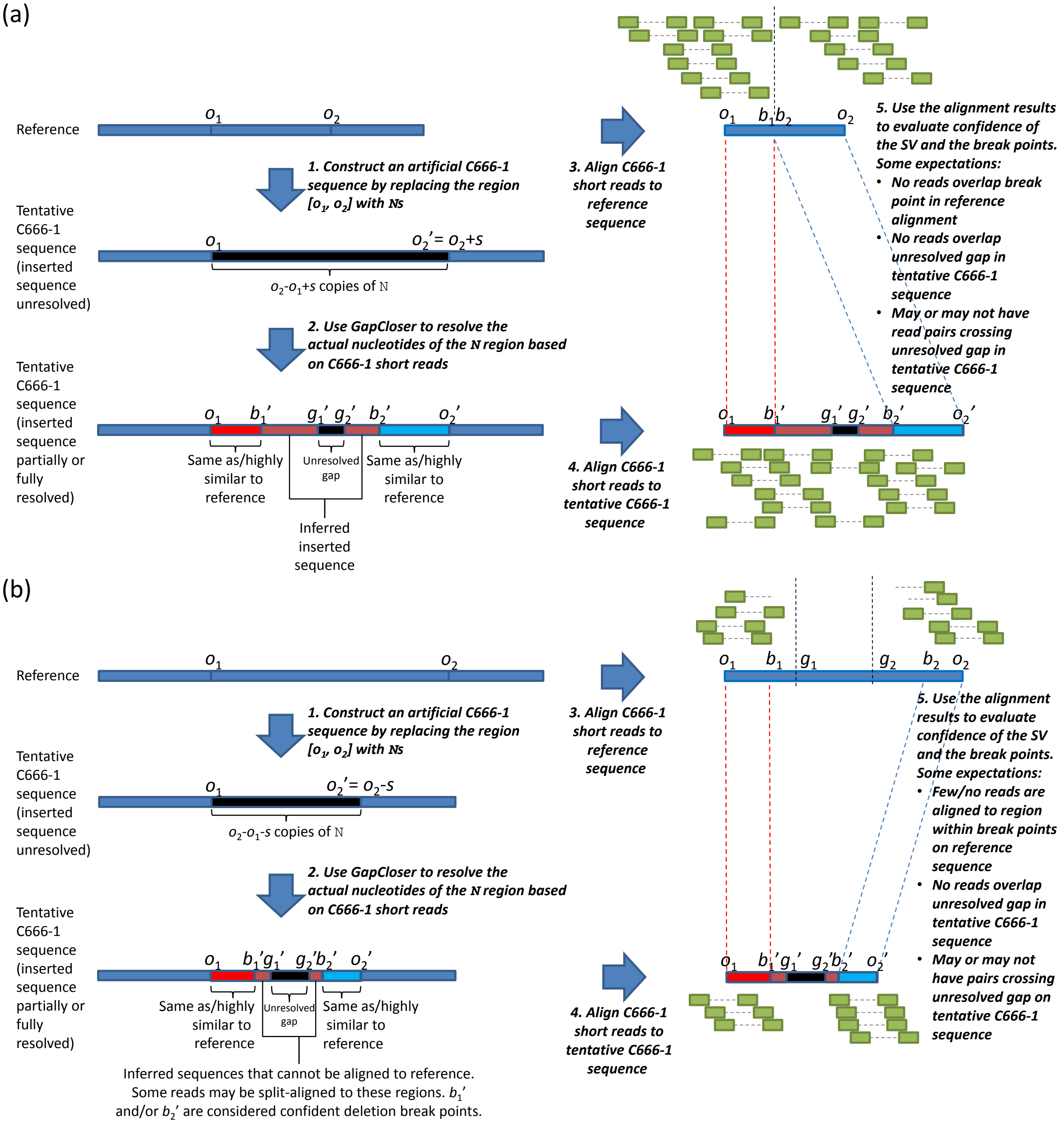

Figure S9 Procedure for combining optical maps and sequencing reads to identify precise SV break points for insertions (a) and deletions (b), and determine the inserted sequences in the case of insertions. o_1 and o_2 represent the rough SV break points on the reference identified by OMSV from optical maps. b_1 and b_2 are the corresponding more precise break points after incorporating sequencing reads, which theoretically should be equal to each other for insertions, but practically there could be a small distance between them due to imperfect resolution of the break points. o_2', b_1' and b_2' mark the corresponding locations of o_2, b_1 and b_2 on the C666-1 sequence after introducing the SV. There may or may not be an unresolved gap (between g_1' and g_2'), depending on whether the whole sequence from o_1 to o_2' can be resolved by the local sequence assembly.

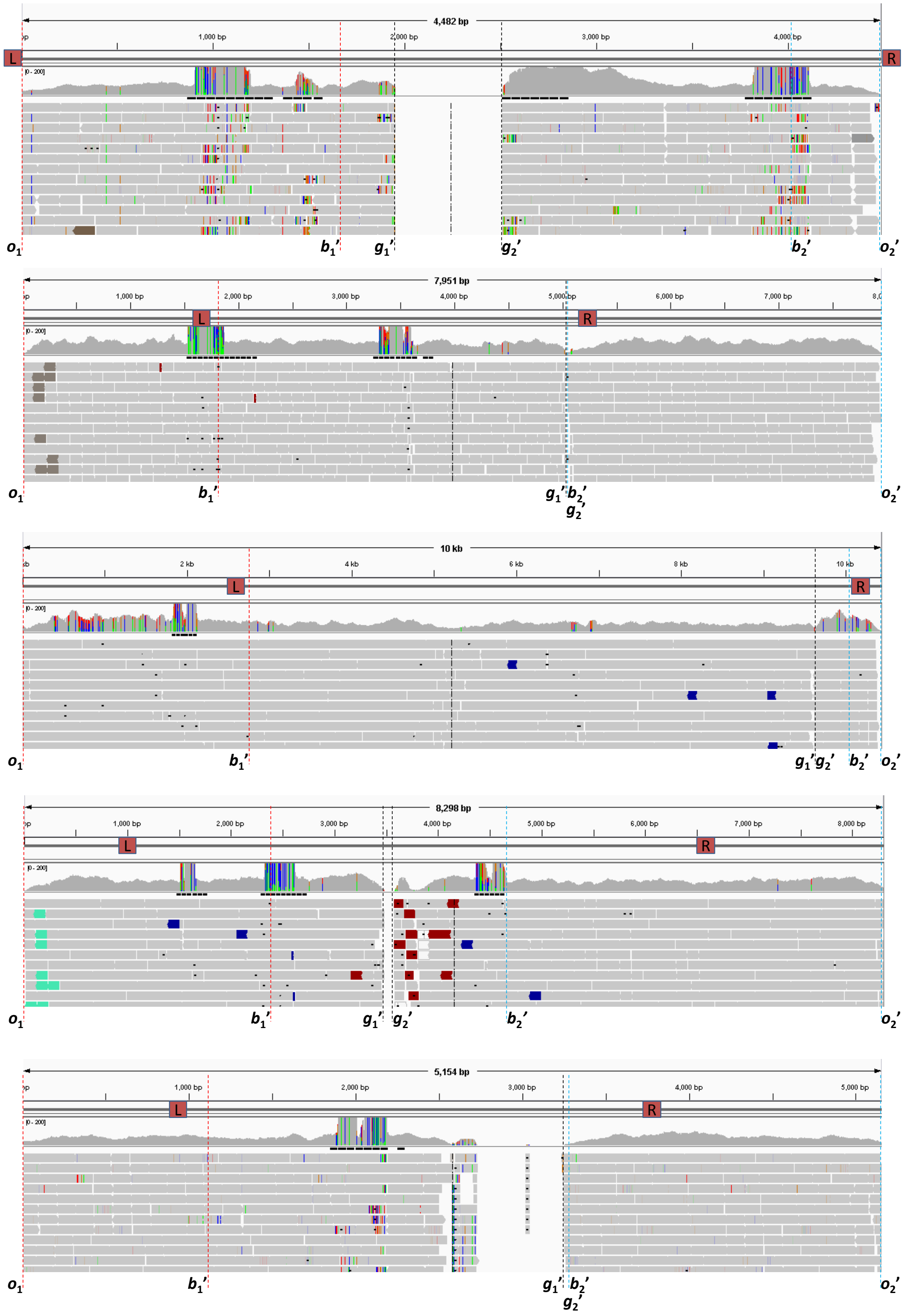

Figure S10 Alignment of sequencing reads to the inferred C666-1 sequences of SVs I_{o1},I_{o4}-I_{o7}. The L and R boxes mark the primer locations. Definitions of o_1, o_2', b_1', b_2', g_1' and g_2' are given in Figure S9. Sequencing read alignments are visualized by IGV. Some high-coverage regions with mismatches in the read alignments are repeat elements.

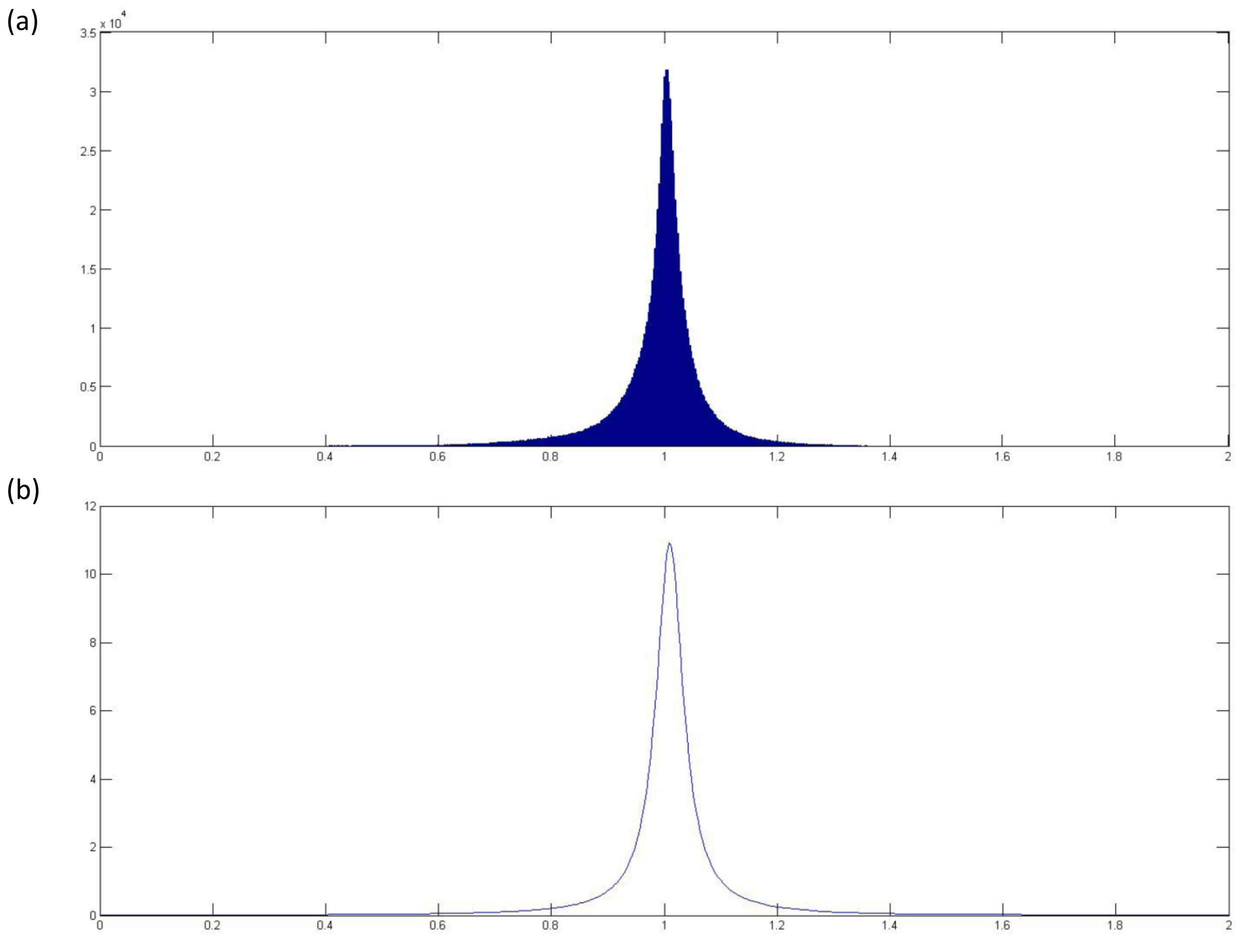

Figure S11 Ratio of the distance between two nicking site labels on an optical map and the corresponding aligned nicking sites on the reference. (a) The distribution of distance ratios observed in the trio OM data. (b) The Cauchy distribution with the position and scale parameters estimated from the trio OM data.
