# Supplementary material for: OMSV enables accurate and comprehensive identification of large structural variations from nanochannel-based single-molecule optical maps
Source: Genome Biol. 2017 Dec 1;18:230. doi: 10.1186/s13059-017-1356-2 (PMC5709945; doi:10.1186/s13059-017-1356-2)

Visualizations of selected C666-1 SVs

Inter-Translocation

Chr5:77835508-Chr8:27558579

Molecule 1

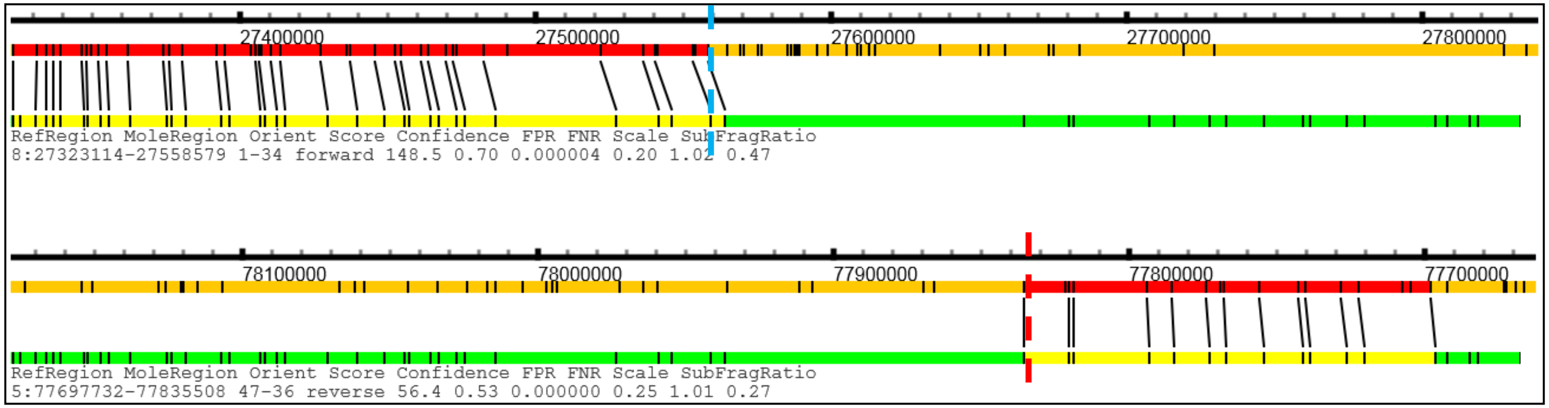

Molecule 2

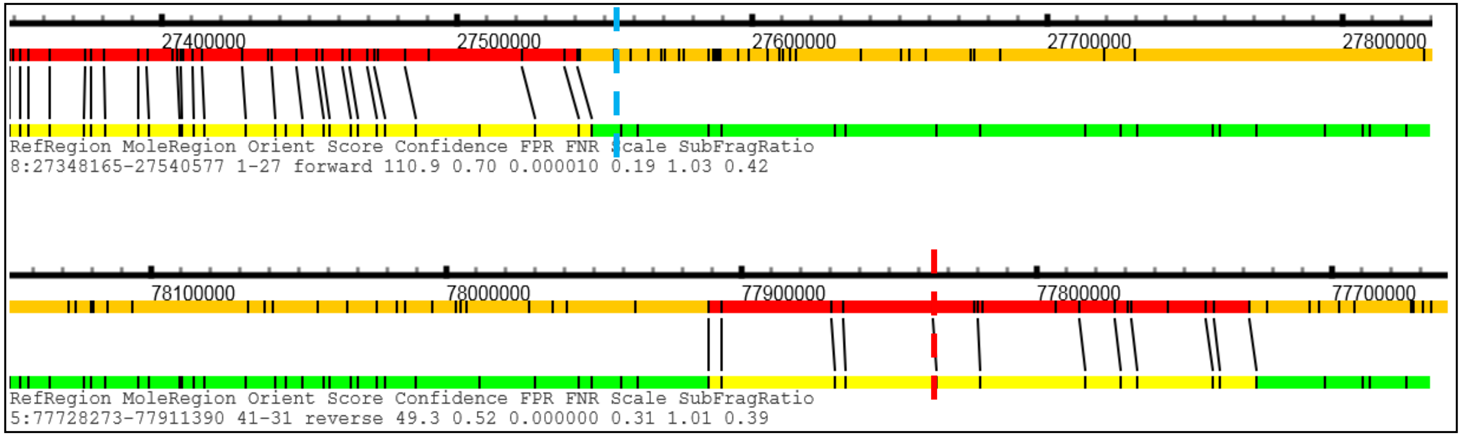

Inter-Translocation
Chr8:102498553-chr16:49600219
(UBR5-ZNF423 translocation)

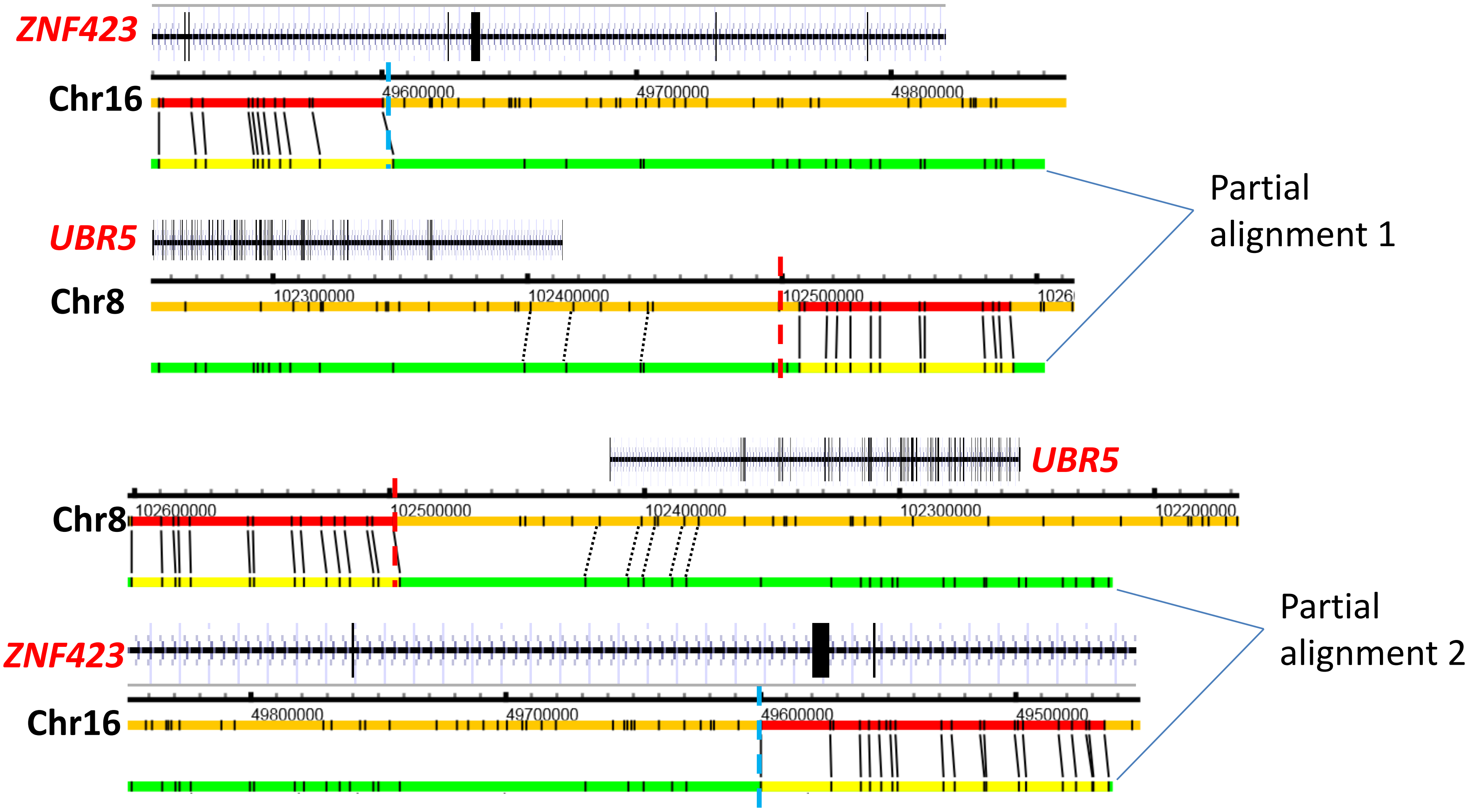

Intra-Translocation
Chr8:22574642-Chr8:30293029

Molecule 1

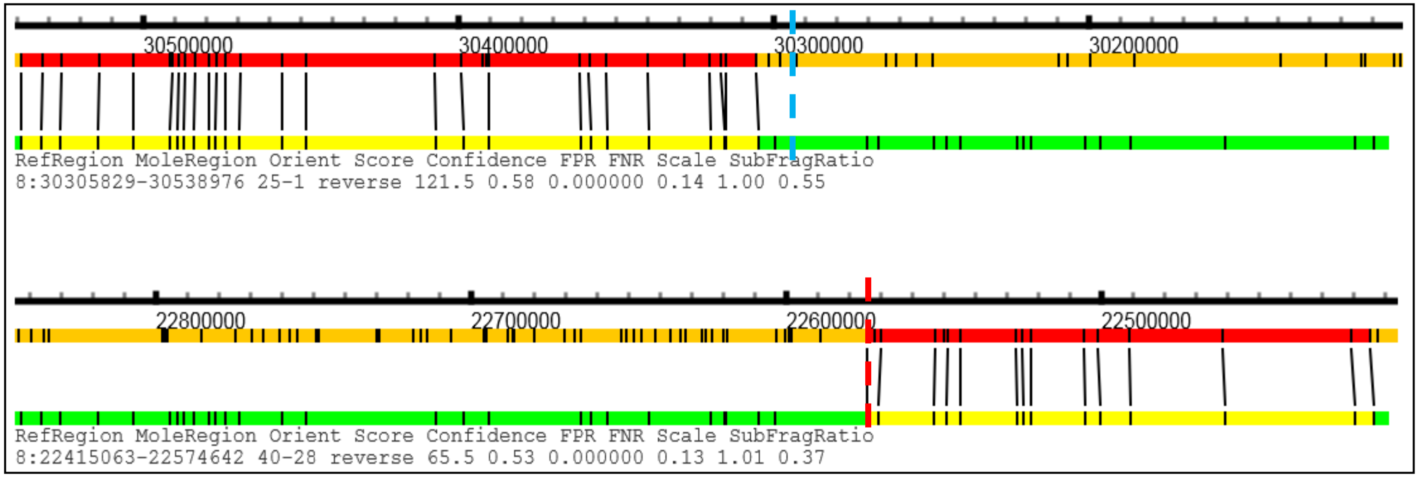

Molecule 2

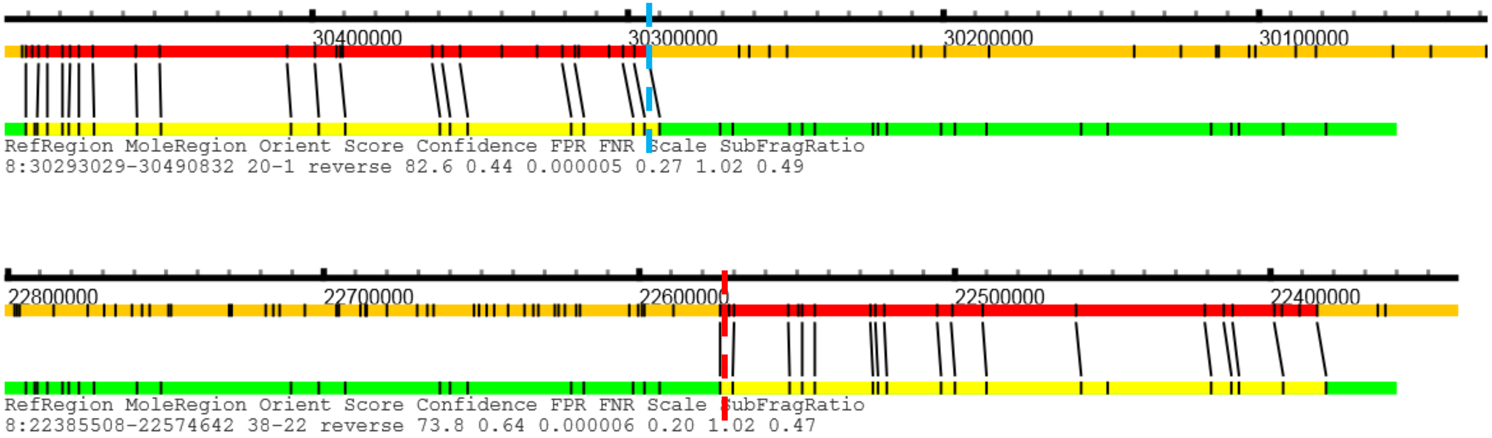

Large Inversion

Chr8:2256826-2433948

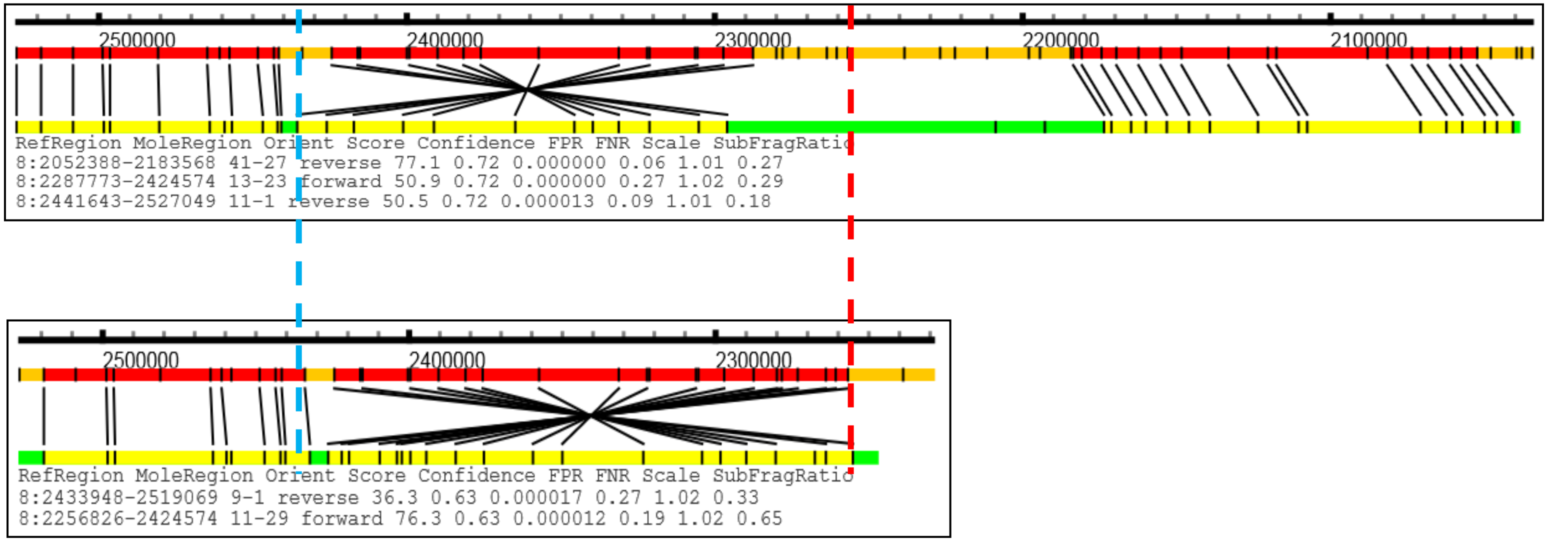

Large Inversion

Chr12:55174173-55516214

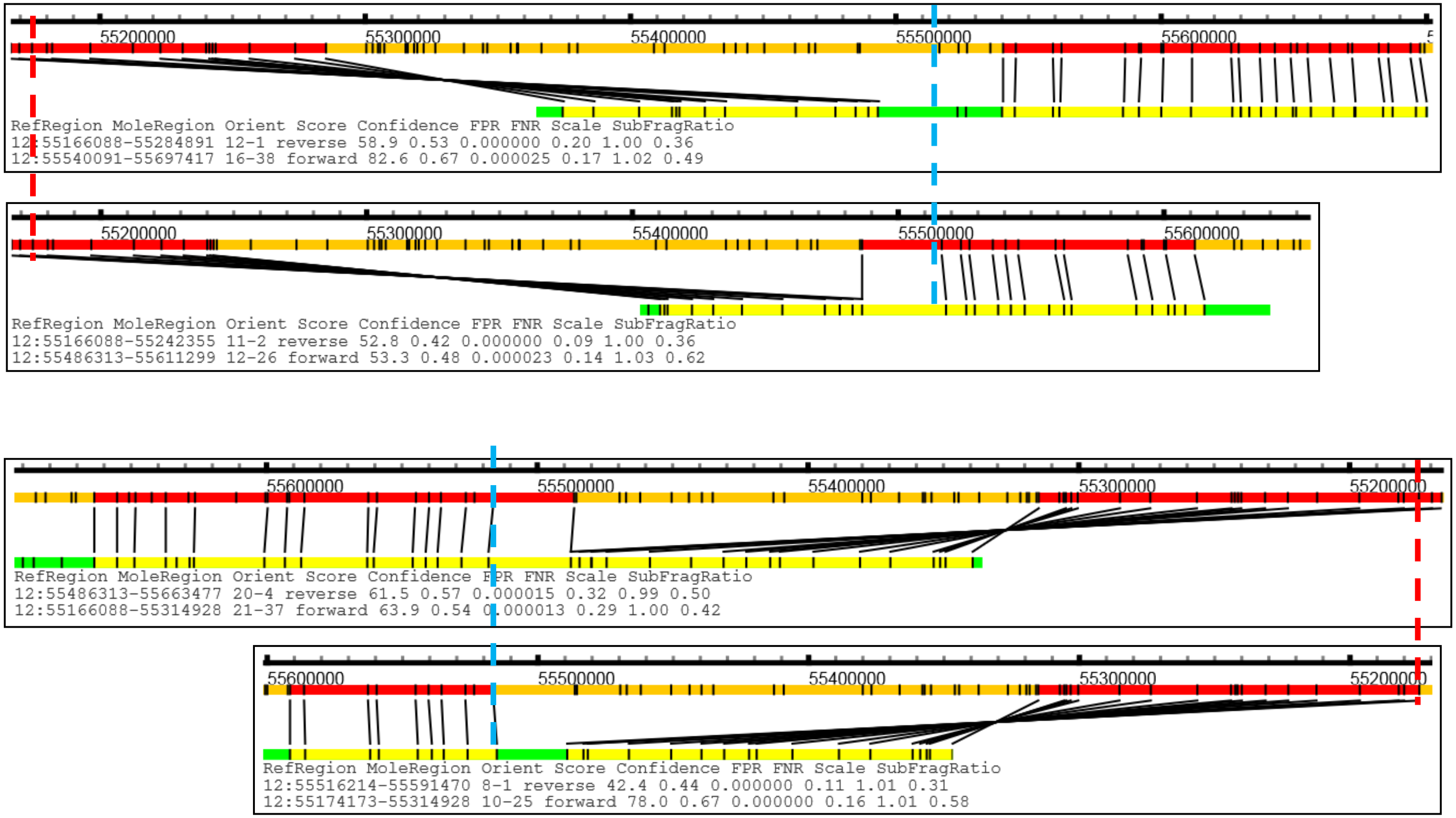

CNV
Chr3: 52231200-52402000

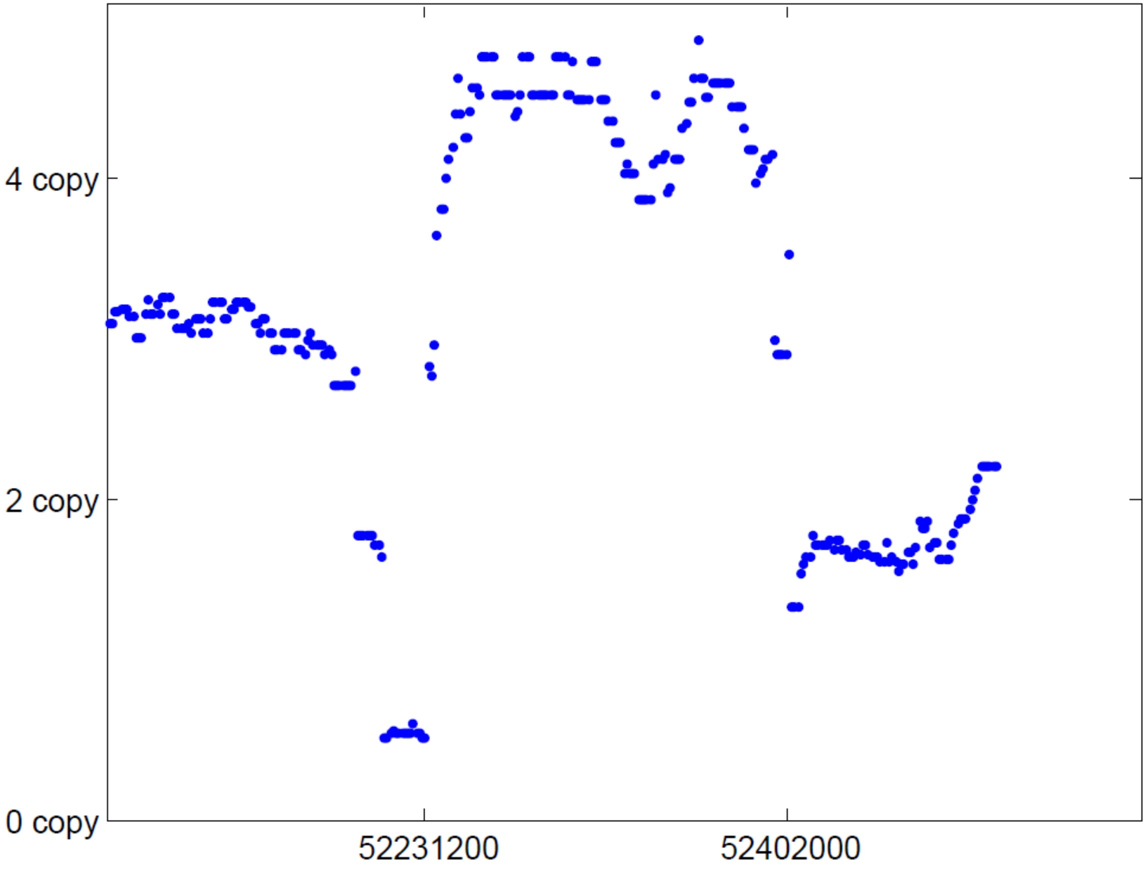

Supplement: Supplementary file 5 — Case studies of complex SVs of C666-1. This file provides visualizations of selected cases of complex SVs identified by OMSV from C666-1. (PDF 487 kb) [file 13059_2017_1356_MOESM5_ESM.pdf]
